# Slow synaptic plasticity from the hippocampus underlies gradual mapping and fragmentation of novel spaces by grid cells

**DOI:** 10.1101/2025.07.30.667696

**Published:** 2025-07-30

**Authors:** Lujia Chen, Ling Liang Dong, Hoon Shin, Farid Shahid, Taylor Malone, Yan Ma, Sreerag Othayoth Vasu, Nai-Wen Tien, Kyle Cekada, Lucy Anderson, Sarthak Chandra, Ila Fiete, Veronica A. Alvarez, Yi Gu

**Affiliations:** 1 Spatial Navigation and Memory Unit, National Institute of Neurological Disorders and Stroke, National Institutes of Health, Bethesda, MD 20892, USA; 2 Department of Brain and Cognitive Sciences and McGovern Institute, Massachusetts Institute of Technology, Cambridge, MA 02139, USA; 3 Laboratory on Neurobiology of Compulsive Behaviors, National Institutes of Mental Health, NIH, Bethesda, MD 20892, USA; 4 Current address: Department of Ophthalmology and Visual Sciences, Washington University School of Medicine, Saint Louis, Missouri 63110, USA; 5 Equal contribution

## Abstract

Animals construct internal “cognitive maps” of the world during navigation in spatial and non-spatial domains, with grid cells in the medial entorhinal cortex (MEC) playing a key role. This requires associating internal position estimates with external cues to reduce spatial uncertainty over time. However, how grid cell representations evolve in novel spaces to support map formation is unclear. To address this question, we longitudinally record grid cells with two-photon calcium imaging over 10 days as mice learn operant tasks in novel virtual linear tracks. We observe that spatial tuning of grid cells is present immediately in novel tracks but evolves as a significant fraction of spatial fields shift backward on a run-by-run basis, within and across days. Backward shifts are more prevalent and persistent in successful learners. The fields gradually stabilize across days, anchored by landmarks, suggesting a slow plasticity mechanism that results in an increasingly fragmented and stable map. The backward shifts partially reset daily, reflecting a slower consolidation timescale. We show that though individual fields of a cell shift differentially, co-active fields of co-modular grid cells shift together, indicating their coupled dynamics keep them on the same two-dimensional torus during this plastic period. Next, we build a simple entorhinal-hippocampal model that explains the diverse phenomena - grid field shifts, fragmentation, and increasing fidelity of the spatial map - and predicts slow Hebbian plasticity in the return hippocampal-to-entorhinal pathway. Finally, using *ex vivo* slice electrophysiology, we show that plasticity in an indirect hippocampus-to-MEC pathway correlates with spatial learning performance and could account for the hypothesized slow plasticity of the model. Together, our study provides multifaceted evidence of slow plasticity in synapses from the hippocampus to the MEC, elucidating the formation of stable and fragmented maps that combine internal and cue-driven positional estimates in rich environments, elucidating cognitive map formation during spatial learning.

## Introduction

The ability of animals to form an internal “cognitive map” of an environment based on cues such as landmarks and boundaries is critical for navigating complex landscapes^1^. In mammals, this map is primarily represented by spatially modulated cells in the hippocampus and entorhinal cortex^2^. Recent studies show that the same cells form cognitive maps of non-spatial domains^3,4^. Key components of the map include place cells in the hippocampus^5^ and grid cells in the medial entorhinal cortex (MEC)^6^, with place cells active at one or a few locations, and grid cells forming a regular tiling pattern in open arenas. In more complex environments, grid cells fragment the space by forming a number of regular local maps with discontinuities between them^7^.

We focus on two key questions: How are maps, which involve associating internal positional estimates with external cues, formed? What is the role and dynamics of grid cells in this process? It is known that place cells^8–10^ and grid cells^6,11^ quickly exhibit spatially tuned responses in novel environments, with behavioral time scale synaptic plasticity (BTSP) playing a role in rapid place field emergence^12^. In grid cells, the rapid appearance of fields in novel environments can be attributed to their internal generation dynamics^13–15^, which exists even during sleep^16–18^, and by cue-driven inputs^11^ and reward locations^19,20^. On a very long time-scale (weeks to month), place fields appear and disappear but their combined responses still encode spatial information about the environment^12,21–24^.

**Figure 1:**
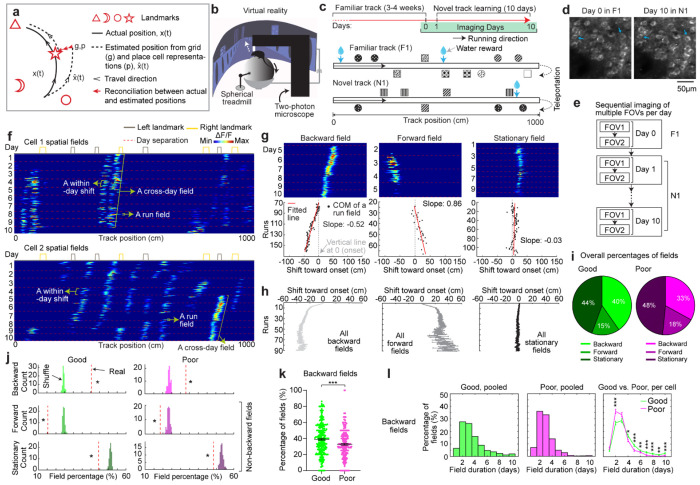
MEC grid cells display activity fields with continuous run-by-run shifts across multiple days **a.** Schematic of forming accurate internal spatial map by grid and place cells for navigation via spatial learning. During spatial learning, internal representation of external environment and estimation of movement are corrected by external landmarks. **b.** Two-photon imaging setup, adapted from Malone et al. 2024^38^. **c.** Experiment design. Top: experiment schedule. Mice perform 10 days novel track (N1) learning after training in a familiar track (F1). Bottom: one-dimensional virtual reality (VR) design of the familiar and novel tracks, adapted from Malone et al. 2024^38^. **d.** Example of the same field of view (FOV) in F1 and day 10 in N1, adapted from Malone et al. 2024^38^. **e.** Imaging workflow. One or multiple FOVs were imaged for one mouse per day in the same order. **f.** Example calcium dynamics of cross-day fields of two grid cells. **g.** Examples of three types of cross-day field (backward, forward, stationary). For each type, top: calcium dynamics of a fields; bottom: centers of mass (COMs) of run fields with their linear regression results. **h.** Averaged trajectories of all the cross-day fields in the three shift types. **i.** Percentage of three cross-day field types, calculated by pooling all cross-day fields of the same shifting type across grid cells and FOVs together. **j.** Comparison of the percentage of three cross-day field types with percentage distribution of run-shuffled fields. **k.** Percentage of backward fields per cell for good and poor performers. **l.** Comparison of cross-day field duration (days) between good and poor performers. Left and Middle: duration distribution of backward fields of good and poor performers. Right: Comparison of the percentage of backward fields with different durations per cell between good and poor performers. *p ≤ 0.05 or the original fraction significantly higher than shuffled fraction in **j**, **p ≤ 0.01, ***p ≤ 0.001, n.s. p > 0.05. Statistical test results are listed in Table S1. Error bars represent mean ± SEM.

The timescales of hours to few days, where actual mapping occurs – the bringing of external cue-driven responses into register with internally inferred positional estimates so that they can be used to correct each other^25–28^ (Fig. 1a) – is our focus here. Over this timescale in open fields, the initially less consistent and expanded grid field responses gradually refine and become more regular^29–31^. On one-dimensional (1D) tracks traversed in one direction, place fields shift backwards with changes in field skewness and width^24,32–37^. In other words, the representations in both entorhinal cortex and hippocampus gradually adjust during map acquisition, but it is unclear how they might relate and what plasticity mechanisms drive this refinement.

Here, we seek a deeper understanding of how map learning unfolds in the brain. To this end, we conducted longitudinal two-photon calcium imaging in the MEC as mice learned novel virtual linear tracks containing local cues over a period of 10 days. The cues served as strong anchoring landmarks that led to the formation of a fragmented grid cell map. We discovered that, as seen earlier for place cells^24,32,34,37^, grid fields gradually shifted backward on a run-by-run basis with a gradual consolidation process, in which each day’s learning was only partially retained. This finding raises the possibility that grid cell shift might drive or be driven by place cell shift; however, in grid cells, the backward shift is interpretable as revealing a growing map fragmentation in which a consistent map is used within inter-cue intervals but the maps between cues are increasingly different.

We built an entorhinal-hippocampal model that captures diverse phenomena and predicts that backward shifts are shaped by temporally delayed Hebbian plasticity in a projection from cue-driven hippocampal cells to grid cells. Using *ex vivo* slice electrophysiology, we found that plasticity in an indirect synaptic pathway from the hippocampus to grid cells was correlated with spatial learning performance, consistent with the predicted temporally delayed Hebbian plasticity pathway of the model. These combined approaches provide multifaceted evidence for a Hebbian plasticity-based mechanism in synapses from the hippocampus to the MEC that gradually creates a spatially stable fragmented grid map anchored to salient environmental features. This work offers new insights into how the brain integrates internal and external information during spatial learning to construct flexible representations, a core function of spatial cognition.

## Results

### Grid fields continuously shift to earlier positions within and across days

**Extended Data Figure 1.**
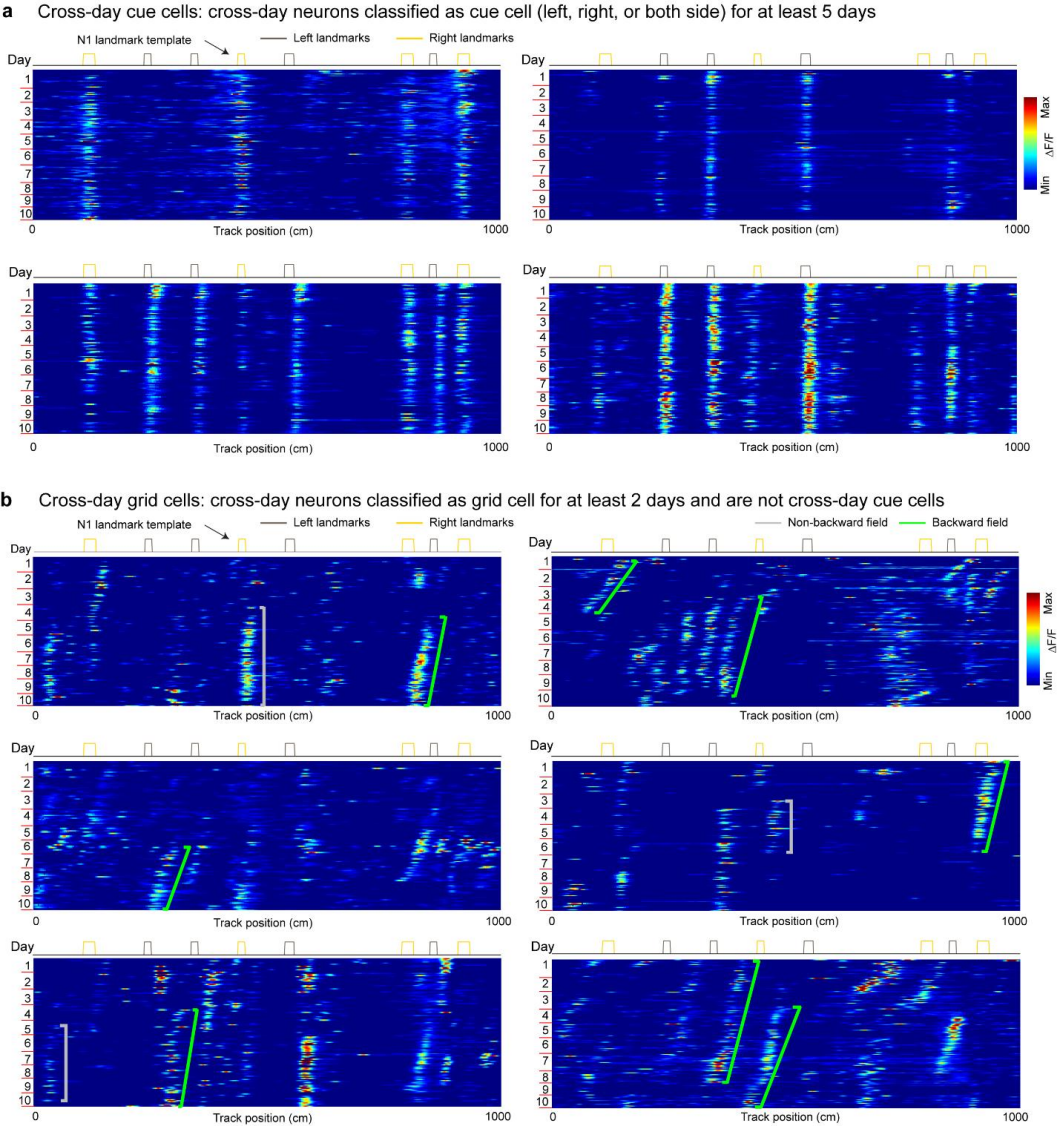
Example cross-day cue cells and cross-day grid cells, related to Figure 1 **a.** Example cross-day cue cells consistently responsive to right landmarks, left landmarks, and landmarks on both sides of the track across all learning days. **b.** Example cross-day grid cells displaying cross-day shifts.

To characterize grid cell activity over extended spatial learning, we analyzed MEC activity in GP5.3 mice^38^ unidirectionally navigating virtual reality (VR) linear tracks for multiple runs each day, which stably expressed a fluorescent calcium indicator GCaMP6f in excitatory neurons in layer 2 of the MEC^38–40^ (Fig. 1b). The mice first explored a track until familiarization (familiar track, F1), then underwent 10-day spatial learning on a novel track (N1) featuring a reward and eight new landmarks at different locations compared to F1 (Fig. 1c). We tracked calcium dynamics in the same group of MEC cells from F1 to day 10 of N1 (Fig. 1d). Each day, 1-3 fields of view (FOVs) in each mouse were imaged in the same order (Fig. 1e). Of the 15 participating mice, 11 were classified as good performers and 4 as poor performers based on their reward-predictive behaviors on N1, indicating successful and unsuccessful spatial learning, respectively^38^. Neurons were classified as grid cells based on reliable spatial field patterns (a field is defined by elevated run-averaged calcium responses above chance in adjacent spatial bins^38^) on at least two days without conjunctive cue activity, defined as having responses locked to multiple landmarks^41,42^ (Extended Data Fig.1a). We focused on grid cells active across the 10 days of N1 learning.

**Extended Data Figure 2.**
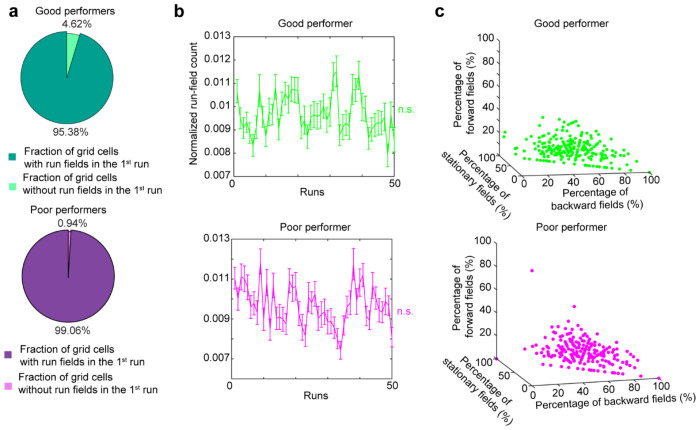
Grid cells display activity immediately upon exploring the novel environment and cross-day fields of different types, related to Figure 1 **a.** Percentage of grid cells recorded in the first field of view (FOV1) that display run field in the first run of the first novel environment learning day. **b.** Normalized run field count of first 50 laps for grid cells in FOV1. For first and last run, the number of fields were normalized by the track area with imaging data, for all remaining runs the number of fields were normalized by track length. **c.** Percentages of cross-day fields with different shift types for individual grid cells recorded in good and poor performers. *p ≤ 0.05, **p ≤ 0.01, ***p ≤ 0.001, n.s. p > 0.05. Statistical test results are listed in Table S1. Error bars represent mean ± SEM.

**Extended Data Figure 3.**
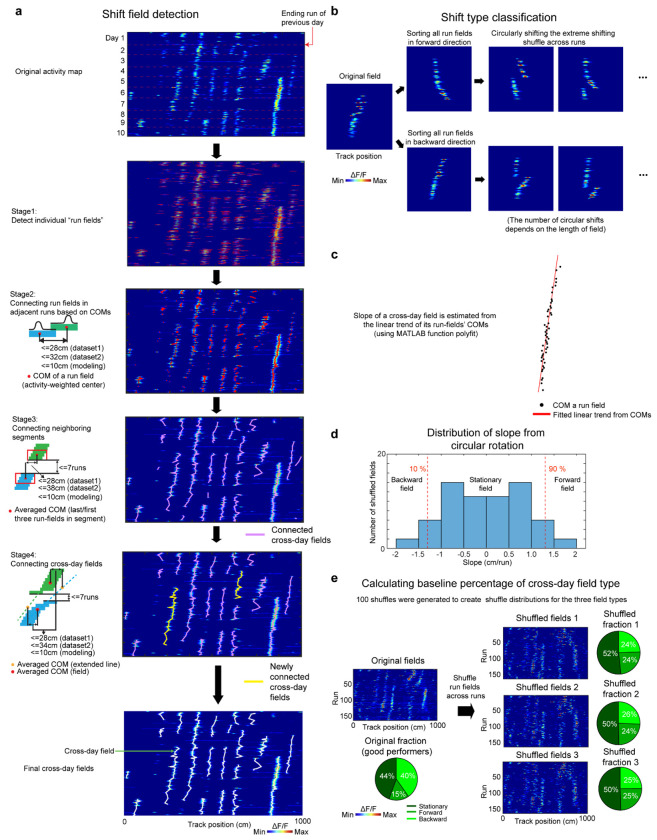
Schematic of cross-day field detection and shifting type classification, related to Figure 1 **a.** Schematic of cross-day field detection. After run field detection, cross-day fields were identified by first connecting closest run fields in adjacent runs, then connecting neighboring run field segments, and lastly connecting close-by cross-day fields sharing a similar trend and with averaged run field center of masses (COMs) at nearby track positions. **b.** Schematic of the cross-day field bootstrap shuffling for generating the baseline slope distribution. **c.** Schematic to determine the slope of the cross-day field using the COMs of individual run fields. **d.** An example bootstrap slope distribution used to determine the shifting type of a single cross-day field. All shuffled slopes of one field were pooled to construct a slope distribution, which was used to determine the significance and the direction of the field’s shift. **e.** Schematic of calculating the shuffle percentage for cross-day field types.

We observed that most grid cells exhibited spatial activity in the first run in N1 (Extended Data Fig.2a), and the number of significant spatial fields detected on a run-by-run basis (“run fields”) remained largely stable across runs (Extended Data Fig.2b). These results are consistent with previous observations that grid fields appear immediately upon environmental change^6,11^, supporting the concept of internally generated grid cell dynamics as predicted by attractor models and confirmed by experimental data ^13,16–18^. Remarkably, grid fields appeared to consistently shift backward to earlier track locations on a run-by-run basis within and across days (Fig. 1f and Extended Data Fig.1b), suggesting a gradual adjustment in spatial representation. To characterize this shift, we connected adjacent run fields within and across days (“cross-day fields”) (Extended Data Fig.3a). We determined the shift directions of cross-day fields based on the slope of the best-fit line through their centers of mass (COMs). Backward or forward shifting cross-day fields had negative or positive slopes, respectively (Fig. 1g, h). The significance of the shift was assessed by comparing the slope with bootstrap slopes of circularly shifted run fields (Extended Data Fig.3b–d). This approach revealed cross-day fields that significantly shifted backward (backward fields), forward (forward fields), and those without significant shifts (stationary fields, Fig.1g, h). All three shift types were observed in good and poor performers (Fig. 1i) and the same cells could display fields in different shift types (Extended Data Fig.2c). We then compared the abundance of these field types to random chance by shuffling cross-day fields 100 times within runs and reidentifying their shift features (Extended Data Fig.3e). The percentage of backward fields exceeded random chance, while stationary and forward field occurrences were below random (Fig. 1j). Therefore, we focused on backward fields and combined forward and stationary fields as non-backward fields.

**Extended Data Figure 4.**
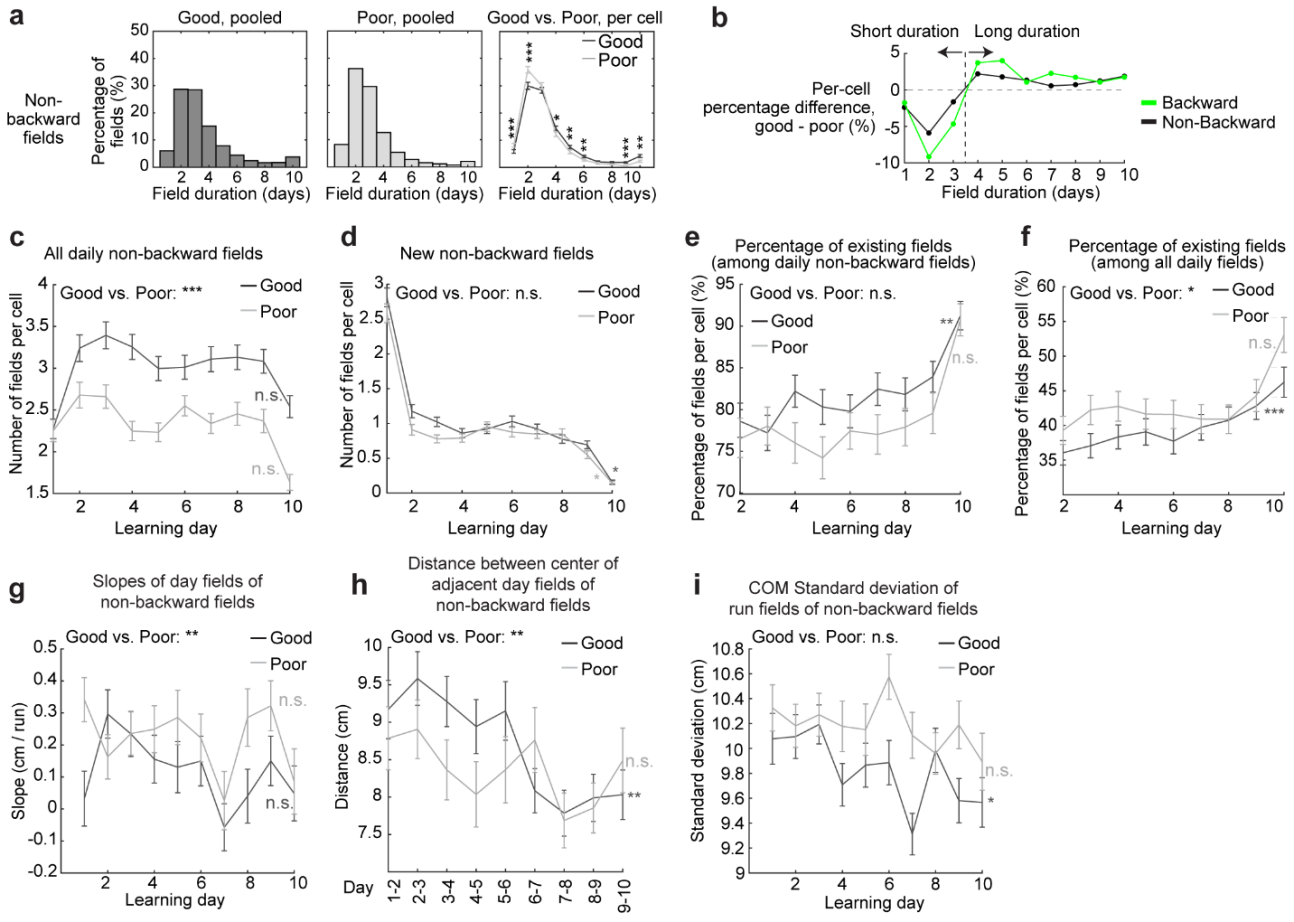
Stabilization analysis for the non-backward fields, related to Figure 1 and 2 **a.** Comparison of cross-day non-backward field duration (days) between good and poor performers. Left and middle: duration distribution of non-backward fields of good and poor performers. Right: Comparison between the percentage of non-backward fields with different durations per cell. **b.** Difference between good and poor performers in the percentages of their backward (green) and non-backward (black) fields with different durations. **c.** Numbers of daily non-backward fields per cell on each learning day. **d.** Number of new non-backward fields per cell appear on each learning day. **e.** Percentage of existing non-backward fields among daily non-backward fields per grid cell across learning days. **f.** Percentage of existing non-backward fields among all daily fields of different shift types per cell across learning days. **g.** Slopes of day fields of non-backward fields across learning days. **h.** Distances between the centers of adjacent day fields of non-backward fields. **i.** Standard deviation of COMs of run fields within day fields, for non-backward fields. *p ≤ 0.05, **p ≤ 0.01, ***p ≤ 0.001, n.s. p > 0.05. Statistical test results are listed in Table S1. Error bars represent mean ± SEM.

We examined the characteristics of backward fields in good and poor performers. Good performers had a higher percentage of backward fields per cell than poor performers (Fig. 1k). Although backward fields persisted for various durations (the number of days a field was observed) in both groups (Fig. 1l, **left and middle**), good performers had relatively fewer short-duration fields (≤ 3 days) and more long-duration fields (> 3 days) on a per-cell basis (Fig. 1l, **right**). These trends were also seen in non-backward fields but were less pronounced (Extended Data Fig.4a and b).

Overall, the significant presence of backward fields reflects dynamic changes in grid cell spatial representation during novel environment learning. The higher abundance and persistence of these fields in good performers suggest that they are associated with successful spatial learning.

### Backward fields gradually stabilize during learning

If backward fields contribute to learning, their spatial activity change should saturate over successful learning to produce a stable spatial representation. We tested this hypothesis by examining several features of the backward fields.

**Figure 2:**
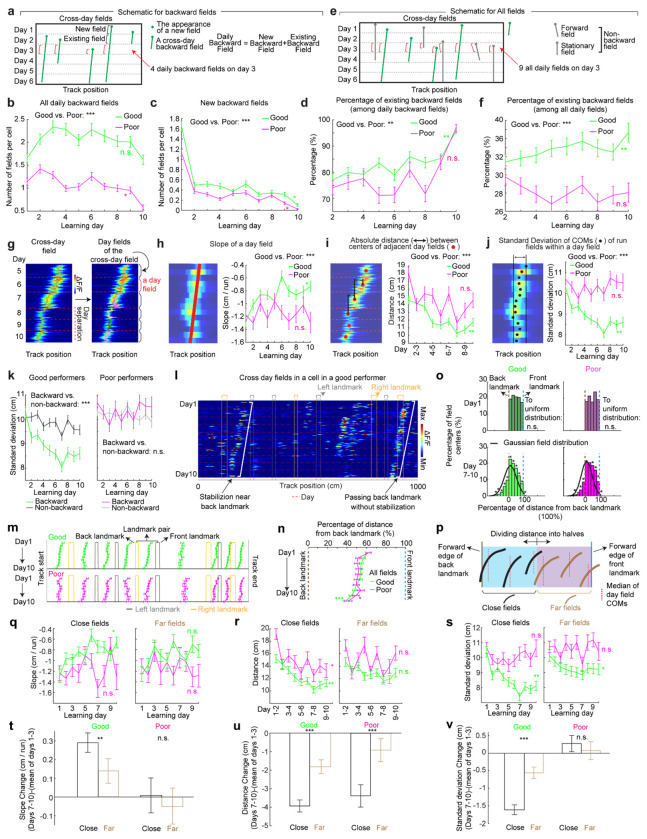
Backward grid fields stabilize and are shaped by landmarks across learning **a.** Schematic for daily, new, and existing backward fields. **b.** Numbers of daily backward fields per cell on each learning day. **c.** Numbers of new backward fields per cell on each learning day. **d.** Percentage of existing backward fields among daily backward fields per cell on each learning day. **e.** Schematic of all day fields, including backward and non-backward fields. **f.** Percentage of existing backward fields among all day fields per cell across learning days. **g.** Schematic of separating cross-day backward fields into day fields. **h.** Slopes of day fields across learning days. **i.** Distance between centers of day fields on adjacent days. **j.** COM standard deviation of run fields within day fields. **k.** Same to **j** but for backward versus non-backward fields in good (left) and poor (right) performers. **l.** A cell containing two backward fields with and without stabilization near landmarks. **m.** Averaged trajectories of backward fields initiated between individual landmark pairs, for good and poor performers. **n.** Averaged trajectories of backward fields initiated between all adjacent landmark pairs for good and poor performers. The distance between each landmark pair was normalized to 100%. **o.** Top: distribution of cross-day fields with their day 1 fields between landmark pairs. Bottom: distribution of day 7-10 fields. The percentages of fields that do or do not cross back landmarks were compared with 20000 simulated percentage values (black), each of which was constructed by the same number of fields that formed a normal distribution around current peak location and covered the same range of track position. **p.** Schematic of defining close and far fields relative to the back landmark. **q-s.** Slopes (**q**) and day field center distances (**r**), and COM standard deviation of run fields within days during learning (**s**), separating close (left) and far (right) fields. **t-v.** Change in **q-s** between days 1-3 and 7-10 for close and far fields. Change is calculated by subtracting the averaged value of day 1-3 from individual values on days 7-10. *p ≤ 0.05 or distance bins significantly different from simulated gaussian distribution (**o** bottom), **p ≤ 0.01, ***p ≤ 0.001, n.s. p > 0.05. Statistical test results are listed in Table S1. Error bars represent mean ± SEM.

We first examined the prevalence of backward fields across learning. In good performers, the number of backward fields per day (these include existing cross-day fields and new fields appeared that day and shifted backward) (Fig. 2a) remained stable during learning (Fig. 2b). The number of new backward fields decreased over days (Fig. 2c), compensated by an increasing fraction of existing backward fields (Fig. 2d). The existing backward fields also increased their fraction among all day fields (backward and non-backward fields) (Fig. 2e and f). In contrast, poor performers had lower fractions of backward fields and no learning-dependent increase (Fig. 2e and f). These results indicate that backward fields exhibit enhanced prevalence and persistence specifically during successful learning. Non-backward fields also improved their persistence, but their prevalence was lower in good performers (Extended Data Fig.4c–f). Thus, the high prevalence of persistent backward fields, but not other field types, is associated with successful learning.

Next, we investigated whether the backward field shift pattern stabilized over learning by dividing each cross-day field by day (“day fields”) (Fig. 2g). In good performers, the per-day negative slope of backward fields gradually approached zero (Fig. 2h). The distance between the COMs of day fields, averaged across run fields within each day, decreased over days (Fig. 2i), and the standard deviation (STD) of run field COMs per day also decreased (Fig. 2j), indicating that backward fields shifted less and reached more consistent locations as learning progressed. In contrast, backward fields in poor performers exhibited persistently negative slopes (Fig. 2h), larger COM distances between days (Fig. 2i) and greater STD of run field COMs per day, without changes over days (Fig. 2j). The stabilization was also observed in the non-backward fields of good but not poor performers (Extended Data Fig.4 g–i). Specifically, the STDs of run field COMs for backward and non-backward fields started at similar levels on day 1, however, the STDs of backward fields decreased more robustly across days, indicating more reliable spatial representation by backward fields post-learning, despite their gradual shifts (Fig. 2k, **left**). This trend was absent in poor performers (Fig. 2k, **right**).

Together, these findings underscore the significant role of backward field stabilization in spatial learning.

### Landmarks shape the trajectories of backward fields during learning

Stable mapping requires that internally derived positional estimates and estimates driven by spatial features reach an equilibrium. Landmarks, which represent key environmental features, have been shown to immediately reshape grid cell activity^11^. We observed that across multiple learning days, as anticipated by backward shifts, backward fields gradually approached the landmarks that came before them (“back landmarks”) and moved away from those ahead (“front landmarks”). Notably, some backward fields near their back landmarks appeared to stabilize near them, while some passed the landmarks without stabilizing (Fig. 2l). To further explore this phenomenon, we investigated how landmarks influenced backward field shift during learning.

We first analyzed field trajectories in relation to adjacent landmarks (Fig. 2m). Backward fields were grouped between each adjacent pair of landmarks, with landmark locations defined by their front edges. The start and end of the track were also considered landmarks due to their similar visual impact. For both good and poor performers, the average trajectories of most field groups gradually shifted toward their back landmarks without crossing them (Fig. 2m). This pattern persisted when all groups were combined (Fig. 2n). On day 1, day fields were evenly distributed between adjacent landmarks. By days 7 to 10, a significant number of fields accumulated near their back landmarks, and fewer fields crossed the landmarks compared to a Gaussian distribution with the same peak location and track coverage (Fig. 2o, bottom, black curve) representing a symmetrical distribution scenario. These results suggest that back landmarks attract backward fields.

**Extended Data Figure 5:**
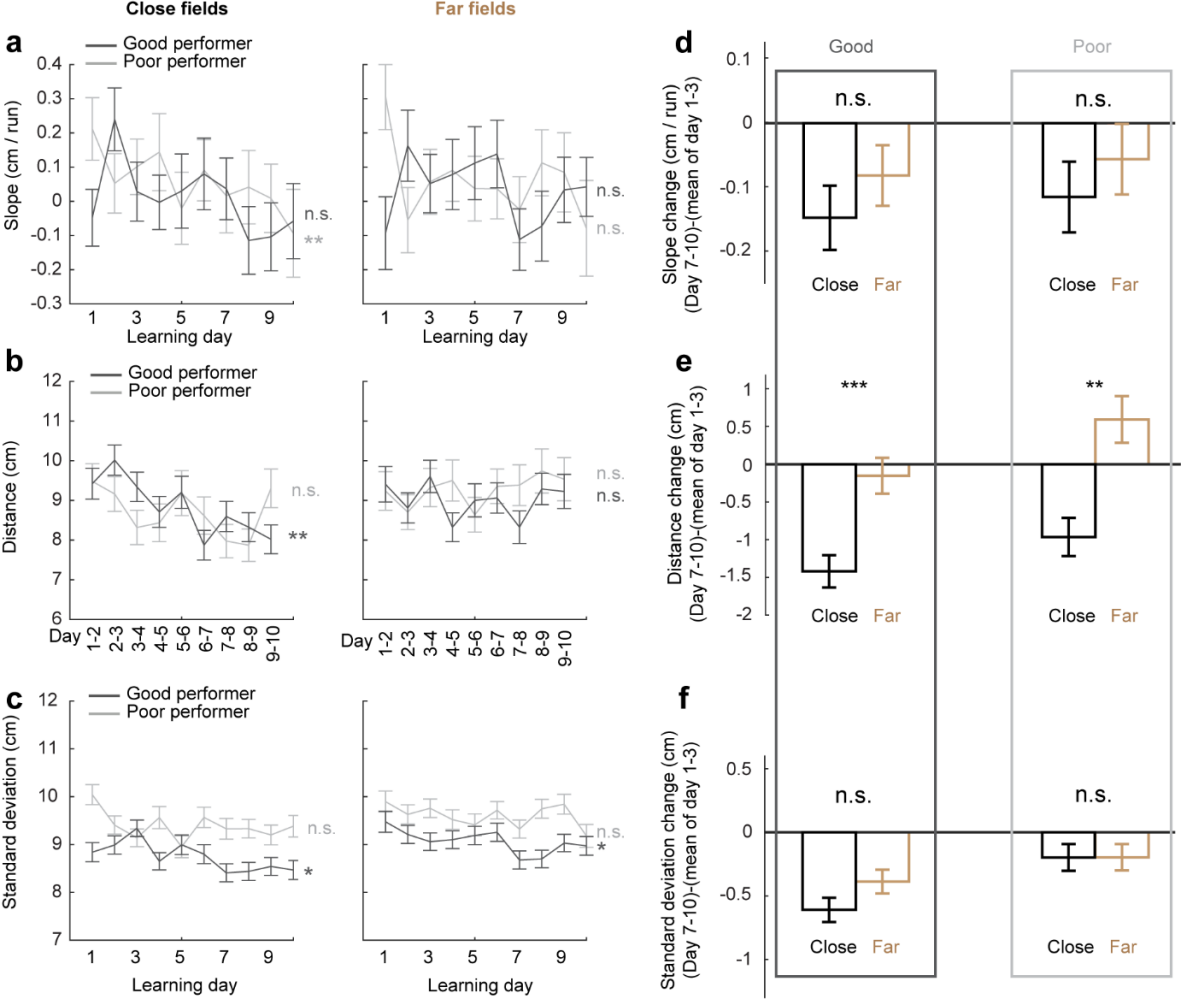
landmarks do not robustly affect the trajectories of cross-day non-backward fields, related to Figure 2 **a.** Slope of day fields across learning days for close and far non-backward fields. **b.** Center distance of adjacent day fields across days for close and far non-backward fields. **c.** COM standard deviation of run fields within day fields across learning days for close and far non-backward fields respectively. **d.** Change in day field slopes between days 1-3 and days 7-10, for close and far fields. **e.** Change in field center distance between days 1-3 and days 7-10, for close and far fields. **f.** Change in COM standard deviation of run fields between days 1-3 and days 7-10, for close and far fields. *p ≤ 0.05, **p ≤ 0.01, ***p ≤ 0.001, n.s. p > 0.05. Statistical test results are listed in Table S1. Error bars represent mean ± SEM.

To further determine if landmarks stabilize nearby fields, we compared stabilization levels of backward fields at varying distances from back landmarks. We divided the distance between each adjacent landmark pairs into halves, categorizing the fields as either “close” or “far” from the back landmark based on the distance from the field location (median of day field COMs) and the back landmark (Fig. 2p), We then combined all close or far fields across adjacent landmark pairs to analyze their stabilization during learning. In good performers, close and far fields exhibited significant differences, with only close fields showing reliable stabilization. Close field slopes approached zero (Fig. 2q), distances between adjacent day field COMs decreased (Fig. 2r), as did the STDs of run field COMs within days (Fig. 2s). Learning-dependent changes in all these parameters, calculated as parameter values on days 7-10 minus the averaged value on days 1-3, were greater for close fields, indicating greater stabilization (Fig. 2t–v). These trends were also observed in poor performers (Fig. 2q–v) and in non-backward fields (Extended Data Fig.5) but were less consistent. These results indicate that during successful learning, back landmarks preferentially stabilize nearby backward fields, whereas fields proximal to front landmarks are less stabilized.

Overall, during learning, back landmarks attract and stabilize nearby backward fields, leading to an over-representation of landmark information.

### Grid field locations partially reset between adjacent learning days

**Figure 3.**
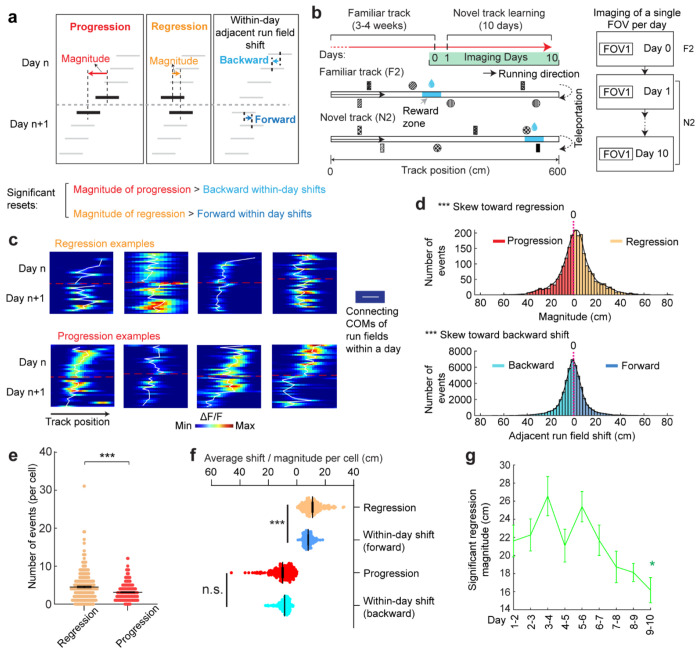
Backward-shifting fields partially reset between adjacent days **a.** Schematic of progression and regression events, and the within-day adjacent run field shifts in the same direction (backward and forward, respectively). The magnitude of a progression or regression event is represented as the shift distance between the COMs of the first run field on day n+1 and the last run field on day n. **b.** Schematic of the experiment design for dataset 2. Left: experiment timeline. After 3-4 weeks of training in a familiar track (middle, F2), mice underwent a 10-day novel track learning (bottom, N2). The mice had to correctly stop within a hidden reward zone (blue) to trigger water reward delivery. Right: imaging schedule. Only one FOV was imaged per day in this experiment, thus no additional online learning was missed in between the two imaging sessions. **c.** Example regression and progression events. **d.** Magnitude distribution of regression and progression events, and within-day adjacent run field shifts in forward and backward directions, combining all backward fields. **e.** Numbers of regression and progression events per cell. **f.** Comparing the distances of regression and progression events to the within-day adjacent run field shift in the corresponding direction. **g.** Magnitudes of significant regression events (above 85^th^ percentile of adjacent run field shifts) across days. *p ≤ 0.05, **p ≤ 0.01, ***p ≤ 0.001, n.s. p > 0.05. Statistical test results are listed in Table S1. Error bars represent mean ± SEM.

On each learning day, the mice experienced the novel track only during their relatively brief VR sessions. We sought to examine the offline dynamics of their grid fields. A fast offline memory consolidation mechanism^43^, or offline predictive plasticity mechanism^43,44^, could manifest as a further backward field shift on the first run of a day relative to the last run on the previous day (“progression”) (Fig. 3a, **left**). Alternatively, if stable memory formation involves a slower consolidation process across many days, the latest learning might not fully persist and would be visible as a forward shift (“regression”) of fields relative to their positions on the previous day (Fig. 3a, **middle**). For offline changes to be statistically significant, their magnitudes should exceed typical adjacent-run field shifts within days (Fig. 3a, **right**).

**Extended Data Figure 6:**
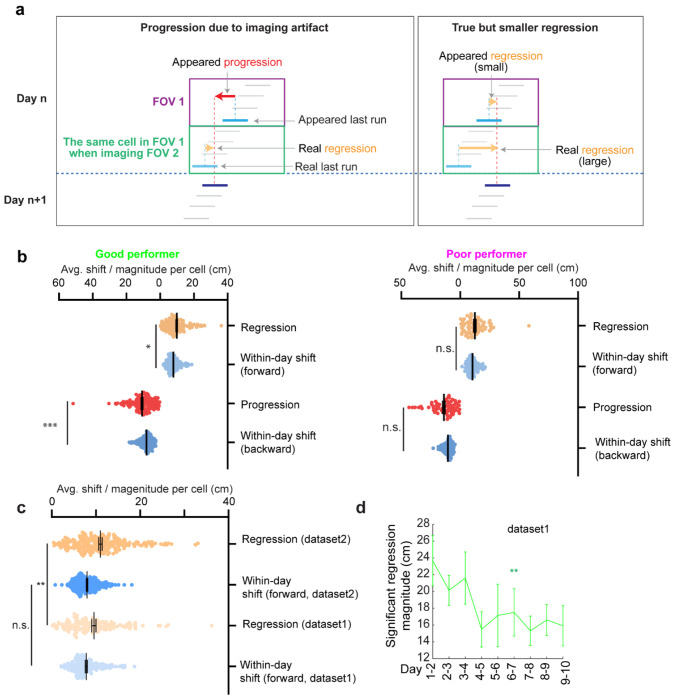
How dataset 1’s imaging scheme affect the investigation of forward reset, related to Figure 3 **a.** Schematic of how the imaging protocol of dataset 1 could affect the magnitudes of progression and regression events, respectively. Left: progression could be artificially created due to the missing data of FOV1 when FOV2 was imaged in the second half of the imaging session. Right: the magnitude of regression could be reduced due to the same reason. **b.** Comparison of the magnitudes of progression and regressions with within-day adjacent run field shifts in the same direction. **c.** The comparison between regression magnitudes in datasets 1 and 2, and the within-day adjacent run field shift in the forward direction, indicating that the regression magnitude was larger in dataset 2 while the within-day field shifts were comparable in the two datasets. **d.** Magnitudes of significant regression during learning in dataset 1. The significant regression is determined as those above 70^th^ percentile of within-day adjacent run field shift. the 70^th^ percentile was used based on the regression-magnitude-scaled percentile of dataset 2 (significant regression percentile for dataset 2: 85^th^, average regression shift of dataset 2: 10.984 cm; average regression magnitude of dataset 1: 9.503 cm. Scaled significant regression percentile for dataset 1 = 85 * (9.503/10.984) = 73.539 ≈ 70). *p ≤ 0.05, **p ≤ 0.01, ***p ≤ 0.001, n.s. p > 0.05. Significant p values are listed in Table S1. Error bars represent mean ± SEM.

Testing offline progression versus regression effects and thus drawing conclusions about the existence of consolidative dynamics requires that we compare field locations just before the offline interval with those right after the interval. However, in dataset 1, multiple FOVs were imaged daily, meaning that animals continued to perform online learning on the same day while imaging other cells (Fig. 1e). This protocol could induce apparent but false progression effects because of continued backward shifting of online not offline dynamics (Extended Data Fig.6a). Nevertheless, backward fields of good performers showed non-negligible regression magnitudes, suggesting the existence of true regression (Extended Data Fig.6b). In contrast, poor performers exhibited no significant regression or progression, consistent with having little online learning to be unlearned during the offline period.

**Extended Data Figure 7:**
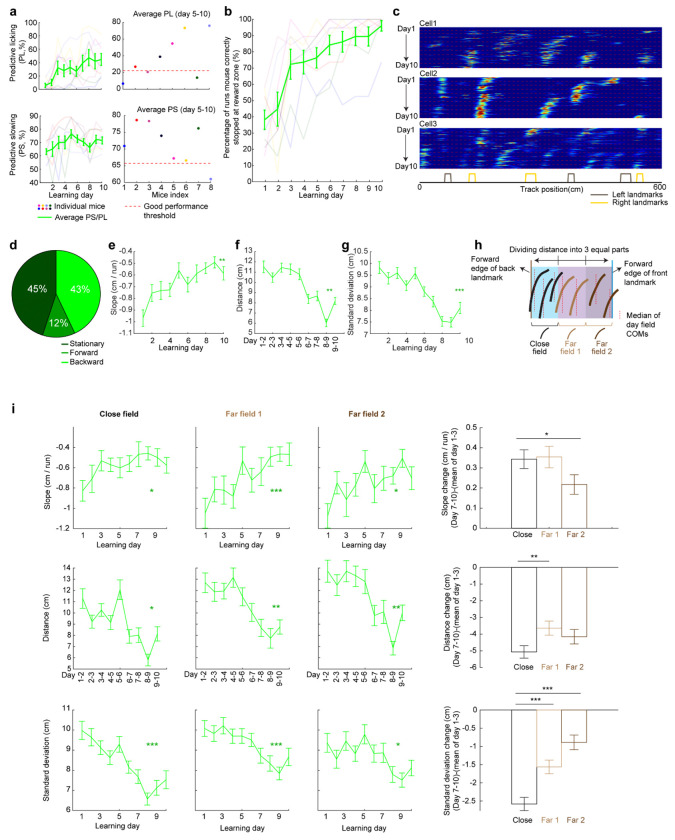
Grid cell dynamics in dataset 2 produce cross-day backward fields similar to dataset 1, related to Figure 3 **a.** Determining good performers in dataset 2. Predictive licking (PL) and predictive slowing (PS) of mice were collected for all the mice during learning. Averaged PS and PL of the last 6 learning days were calculated and compared to the previously reported PS and PL threshold for good performers (PL threshold: 22.2^th^ percentile , PS threshold: 65.7^th^ percentile), as conducted previously^38^. A mouse needed to have both averaged PL and PS below threshold to be classified as poor performers. Otherwise, the mouse was identified as a good performer. Therefore, all mice were classified as good performers. **b.** Percentage of runs when mice stopped within the correct reward zone across learning. **c.** Example cross-day fields of grid cells. **d.** Fraction of shifting types for all cross-day fields. **e.** Slopes of backward day fields across learning days. **f.** Distances between the field centers of backward day fields in adjacent days. **g.** COM standard deviations of run fields within backward day fields across learning days. **h.** Schematic of defining the close and far fields toward the back landmark for dataset 2. Areas between a landmark pair were divided into 3 equal parts, and “close fields”, “far fields 1”, and “far fields 2” were defined if their median field centers fell into specific parts. **i.** Stabilization of close fields, far fields 1, and far fields 2 by landmarks, reflected by their slopes, distances of adjacent day fields, and COM standard deviations of run fields across learning, similar to Fig. 2q–v. *p ≤ 0.05, **p ≤ 0.01, ***p ≤ 0.001, n.s. p > 0.05. Statistical test results are listed in Table S1. Error bars represent mean ± SEM.

We therefore conducted another experiment by continuously imaging calcium dynamics in the same layer 2 MEC neurons over their entire online experience as mice learned a novel track (N2) over 10 days (“dataset 2”, Fig. 3b right). Mice had to stop in a fixed reward zone to receive a water reward (Fig. 3b). This cohort included eight mice, all evaluated as good performers based on the criteria of dataset 1 (Extended Data Fig.7a and b). Similar to dataset 1, we identified grid cells active across the 10 days (Extended Data Fig.7c), with a comparable percentage of backward fields to those in good performers from dataset 1 (Extended Data Fig.7d). The backward fields, especially those close to back landmarks, exhibited obvious gradual stabilization during learning (Extended Data Fig.7e–i). Thus, the grid cell activity in dataset 2 reproduced our observations from the good performers in dataset 1.

Backward fields in dataset 2 exhibited both progression and regression (Fig. 3c), but the magnitude distribution skewed significantly toward regression (Fig. 3d). Regression events per cell outnumbered progression events (Fig. 3e), and regression but not progression magnitudes were significantly larger than the shifts between adjacent run fields in the same direction (Fig. 3f), indicating significant regression, but not progression. As expected, regression magnitudes in dataset 2 were larger than those in dataset 1, while their adjacent run field shift magnitudes within days were comparable (Extended Data Fig. 5c). Across 10 learning days, the magnitudes of significant regression in dataset 2 (exceeding the 85^th^ percentile of adjacent run field shifts) decreased (Fig. 3g), a similar trend observed in dataset 1 (Extended Data Fig. 6d), suggesting a gradual memory consolidation that corresponds to less offline memory loss.

The findings of significant daily regression but not progression suggest that learning-induced plasticity during online periods partially degrades during offline periods. This finding indicates the existence of a separate memory consolidation process, which requires online experience and involves a longer time scale than plasticity induction^45–47^.

### Grid cells exhibit invariant low-dimensional population dynamics and growing map fragmentation over learning

Next, we extended our analysis from the dynamics of individual backward grid fields to the global population dynamics of whole grid modules. We asked whether module activity remains low-dimensional during learning of a new environment when grid fields shift, interact with landmarks, and settle. Though we expect that grid cells maintain a low-dimensional dynamics based on continuous attractor network models^13–15,48^ and demonstrations under many conditions^38,49,50,11,16–18^, it is unclear if they do so during the formation of the spatial map where we have observed that the fields of even a single neuron shift differentially relative to each other (Extended Data Fig.1b and Extended Data Fig.2c).

**Extended Data Figure 8.**
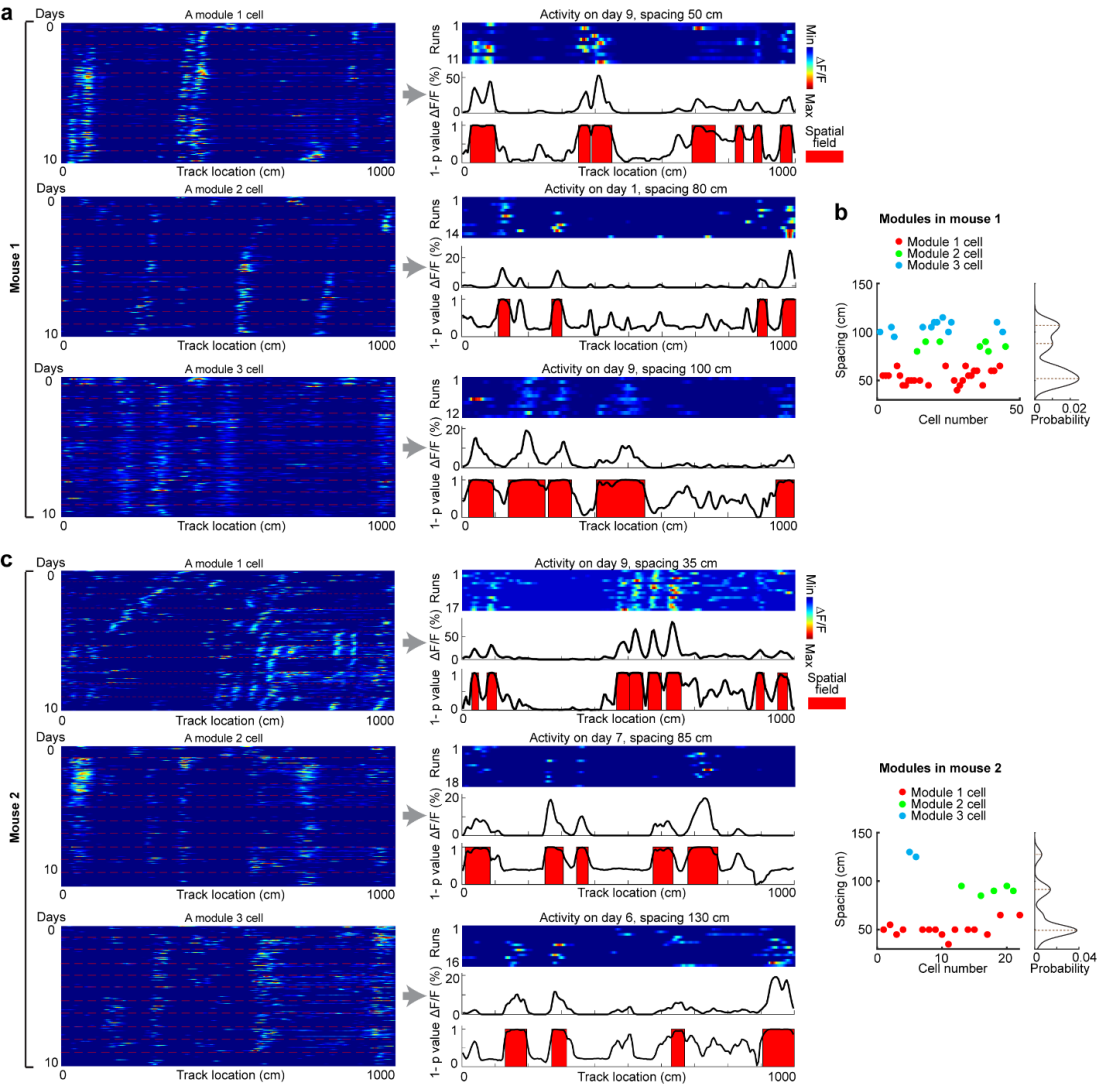
Identification of grid modules, related to Figure 4 Grid modules were identified based on the spacing of the cells within individual days. Since grid fields dynamically changed across days, we inspected the activity of each cell across all days (day 0 in F1 and days 1-10 in N1). The spacing of a cell was determined as its smallest spacing of day fields, which were identified based on the p values of spatially averaged activity (across runs) of the cell within a day in comparison to shuffles^41^. **a.** Example cells for three modules in mouse 1. For each cell: Left: cross-day activity of the cell. Right: activity of the cell on a particular day, during which the cell showed the smallest spacing. Top: run-by-run activity heat plot. Middle: spatially averaged ΔF/F. Bottom: 1-p_value_ of the activity at each spatial bin. Higher 1-p_value_ corresponds to higher activity of the cell compared to shuffle. Spatial fields are highlighted in red. **b.** Grid modules of all cells in mouse 1. This mouse has three modules based on the spacings. **c and d.** Similar to **a** and **b** but for mouse 2.

**Figure 4.**
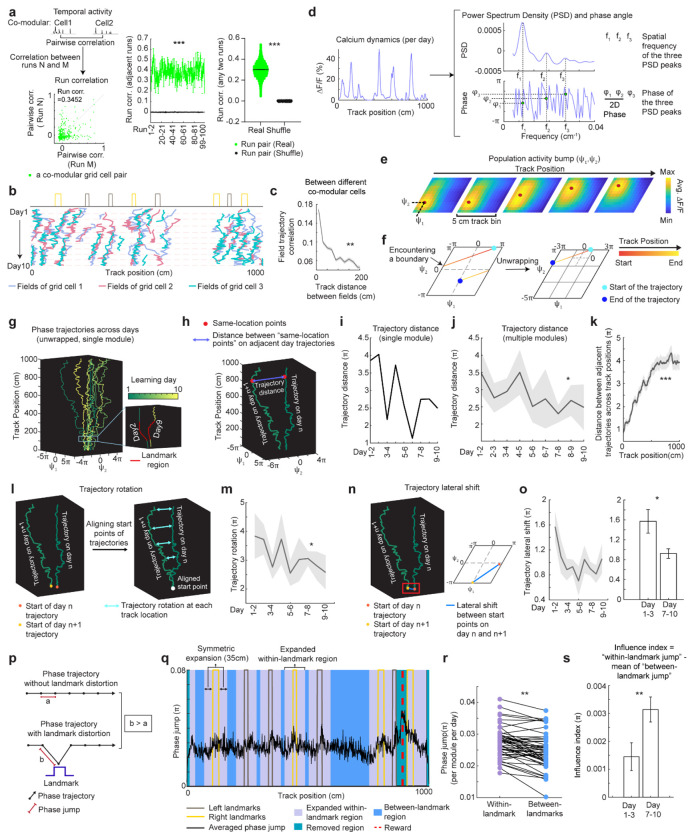
Population dynamics of grid cells over learning remain low-dimensional with collective phase shifts that gradually stabilize **a.** Temporal activity relationships of co-modular grid cells are maintained across runs. Left: the consistency of the relationship was quantified as “run correlation”, which is the correlation between the pairwise correlations of temporal activities of the same co-modular grid cell pairs on different runs (N and M here). Middle: run correlation between adjacent runs for all modules, compared to shuffles. Right: run correlation between all run pairs for all modules, compared to shuffles. **b.** Field trajectories of three co-modular cells, showing similar shift for fields that are spatially adjacent but belong to different cells. **c.** Correlation between cross-day fields from different co-modular grid cells decrease with increased field distance. Correlation is calculated between the field segments of two cross-day fields within the same runs. Correlations were pooled within non-overlapped 20-cm track bins. **d.** Schematic of determining the phase coordinates of grid cells. Left: calcium dynamics of a grid cell, averaged across runs within a day. Right: Power spectrum density (PSD, top) and the corresponding phase (bottom) were calculated from the cell’s calcium dynamics on the left. **e.** Combining phase distribution and calcium dynamics of each cell at individual track locations, an activity bump was revealed and translated within the rhombus. Ψ_1_and Ψ_2_ represent bump coordinates within the phase rhombus. **f.** Unwrapping to solve the issue when the bump trajectory encountered boundaries of the rhombus. Left: an example trajectory encountering boundaries of the rhombus Right: unwrapped trajectory. **g.** Phase trajectories of a group of co-modular grid cells on individual learning days. Right bottom: zoomed in region around the first landmark of the trajectories on two days. **h.** Schematic of for distance calculation between two phase trajectories on adjacent days (trajectory distance). The distance was calculated between each pair of points at the same track location on the two trajectories (“same-location points”). **i.** Trajectory distance for the example in **g**. **j.** Trajectory distance between adjacent days, across all modules. **k.** Trajectory distances of same-location points on adjacent day trajectories from the beginning to the end of the track, combining all modules. **l.** Schematic of trajectory rotation estimation based on trajectory distance between adjacent days. The starts of the two trajectories were aligned to the same point to eliminate the lateral shift. **m.** Averaged trajectory rotation across modules between every adjacent learning day. **n.** Schematic of trajectory lateral shift estimation based on the distance between the starting points of adjacent day trajectories. **o.** Trajectory lateral shift across learning. Left: Averaged trajectory lateral shifts across modules between adjacent learning days. Right: Comparing trajectory shifts on days 1-2, 2-3 versus on days 7-8, 8-9, and 9-10. **p.** Schematic of phase jumps with and without landmark distortion. **q.** Phase jumps along the track. The jumps were averaged across all grid modules and all 10 days. Phase jumps within the first and last 10 cm of the track, as well as those around the reward (863-909 cm), were removed from analysis. **r.** Comparison of phase jumps between within- and between-landmark regions. Each line represents the jumps of one grid module on one day. **s.** Landmark distortion, quantified by “influence index” in early (days 1-3) and late (days 7-10) learning. *p ≤ 0.05, **p ≤ 0.01, ***p ≤ 0.001, n.s. p > 0.05. Statistical test results are listed in Table S1. Error bars represent mean ± SEM.

In good performers from dataset 1, we identified “co-modular” grid cells (cells with similar spacings between nearest fields)^51^ (Extended Data Fig.8), and examined whether these cells exhibited preserved attractor dynamics across learning^52^, so that population states remained localized on the same 2D toroidal manifold. We found that the temporal activity correlations between simultaneously imaged co-modular grid cell pairs (“pairwise correlation”) were highly preserved across runs (significantly higher than shuffles, Fig. 4a), reflecting stable cell-cell activity relationships over learning^38^.

Thus, we have two seemingly opposing findings – that the fabric of cell-cell relationships is invariant even though firing fields shift differentially relative to each other in single cells, indicating large spatial tuning distortions. These can only be reconciled if the differential field shifts in a cell are shared population-wide across co-modular cells: that is, the entire population state shifts coherently across cells, but differentially relative to the underling physical location of the animal at different locations. Accordingly, we examined the shifting of spatially overlapping fields from different co-modular cells, finding that they shifted in tandem across cells (Fig. 4b). Consistent with this, spatially nearby fields from different co-modular cells (based on median of run field COMs) exhibited high trajectory correlations, while those further apart showed much lower correlations (Fig. 4c). Thus, well-separated fields of even a single cell could shift differentially relative to external space, the co-active fields of different cells shift together, indicating preserved population coherence and low dimensionality coexisting with collective state changes relative to space that correspond to field shifts.

Finally, we considered the mapping from intrinsic low-dimensional population states to the animal’s spatial position, how this mapping changes over learning, and how these changes relate to observed grid field shifts. Given the evidence of preserved low-dimensional population dynamics, we estimated a population phase from simultaneously imaged co-modular grid cells in good performers using the Yoon-Lewallen technique, which is based on Fourier transforms of the 1D spatial responses of individual cells^53^. Specifically, Fourier transform produced Power Spectrum Density (PSD) of each cell with three largest peaks in spatial frequencies f1, f2, and f3 (Fig. 4d, top right). f1, f2, and f3 determine the angle of an 1D slice through the underlying two-dimensional (2D) grid that generates the 1D spatial firing fields. For spatial responses of simultaneously imaged co-modular grid cells that are generated from a common underlying 2D triangular grid, f1, f2, and f3 should be the same for the cells, and the sum of f1 and f2 should equal to f3^53^. The 2D phase (φ_1_, φ_2_) of a cell in a phase rhombus was extracted from the Fourier transformation at the locations of f1 and f2 (Fig. 4d, bottom right). Next, we combined the phases of individual co-modular cells with their evolving activity along the track to obtain a moving population activity bump that traced out a population phase trajectory over the track (represented by Ψ_1_, Ψ_2_) (Fig. 4e). To more easily visualize the trajectory with periodic boundary conditions within the phase rhombus (Fig. 4f, left), we unwrapped the population phases across multiple rhombuses, obtaining a largely unidirectional population phase evolution (Fig. 4f, right).

**Extended Data Figure 9:**
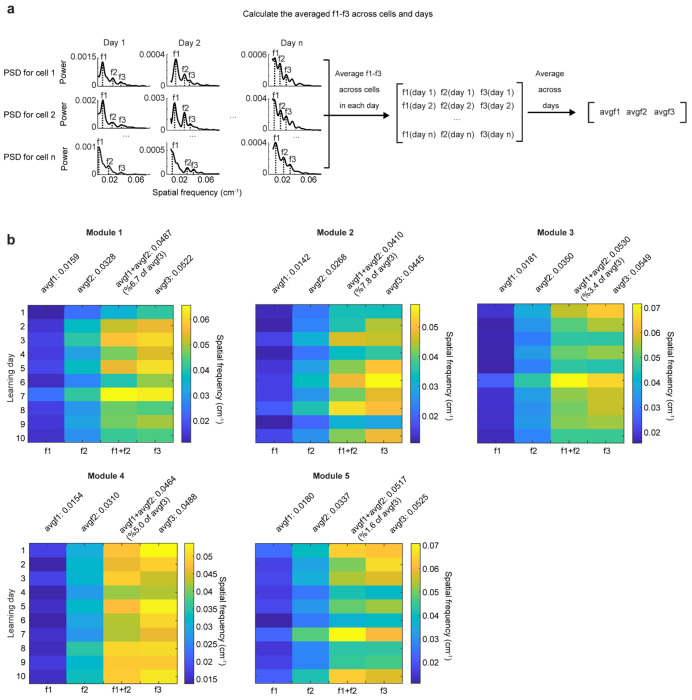
Spatial frequency feature of the selected grid cell modules, related to Figure 4 **a.** Schematic for calculating the avgf1-3 (averaged f1, f2, f3 across days). Within each day, the spatial frequency of the three largest peaks of each co-modular grid cell’s PSD (f1-3) were averaged separately. Following this, the population per day f1-3 were further averaged across days to obtain the final avgf1-3. **b.** Per day f1-3 for the selected five grid cell modules. For all qualified modules, the absolute difference between their avgf1+avgf2 and avgf3 must be smaller than 8% of avgf3.

For precise population dynamics estimation, the above analyses were applied to five simultaneously imaged co-modular cell groups with more than 10 cells, whose sum of averaged f1 and f2 across days was near the averaged f3 (difference < 8% of averaged f3) (Extended Data Fig. 9). We observed that their population phase trajectory changed over learning (Fig. 4g), with the changes decreasing over time. This was quantified via a trajectory distance between adjacent days, defined as the average of the set of point-wise Euclidean distances between the points on the two trajectories at the same track position (Fig. 4h–j). Overall, the phase trajectory changed through rotation above the starting point, reflected by the gradually increasing trajectory distances for the beginning toward the end of the track (Fig. 4k). The rotation decreased over learning (Fig. 4l–m). The small lateral shifts in the starting phase also decreased over learning, with larger shifts across days 1-3 than days 7-10 (Fig. 4n–o). The rotations and lateral shifts of the population phase trajectory as animals run on the same track over learning potentially cause the observed gradual spatial shifts of the grid fields.

Next, we examined how landmarks influence the phase trajectory and shape map fragmentation. If landmarks strongly modify the positional estimate and drive map fragmentation, the population phase should exhibit larger changes (phase jumps) near landmarks than far from them (Fig. 4p). Indeed, phase jumps were bigger around landmarks (Fig. 4q) and near the track end (which contains two landmarks as well as a water reward, known to attract grid fields^19,20^). To isolate influence of landmarks and remove reward and teleportation effects, we excluded regions near the reward and near the start and end, finding that within-landmark jumps were still larger than between-landmark jumps (Fig. 4r). We quantified the evolution of landmark influence over learning by defining an “influence index”: the average within-landmark jump magnitude minus the between-landmark jump per day. Landmark influence on late days (days 7-10) was higher than on early days (days 1-3) (Fig. 4s), suggesting a growing map fragmentation.

These results show that over learning, the internal population state traces a trajectory on an invariant 2D torus. This trajectory gradually rotates and shifts, and it also exhibits discontinuities or phase jumps at landmarks. The rotations and shifts decrease over learning, indicating a stabilization of the map, while the discontinuities at the landmarks are greater on later days than earlier days, meaning that learning resulted in a more fragmented map.

### A mechanisic model of map learning captures diverse phenomena and predicts delayed hippocampal-to-entorhinal plasticity

To model the mechanisms underlying the above grid cell dynamics, we turned to a theoretical neural circuit model of the entorhinal-hippocampal network and considered the role of experience-dependent plasticity in gradually modifying the internal and cue-driven dynamics of the grid cell response. Our model builds on the theoretical Vector-HaSH framework^54^, extending the model by generalizing it to continuous space and modeling slow plasticity in the synapses from the hippocampus to grid cells.

**Figure 5.**
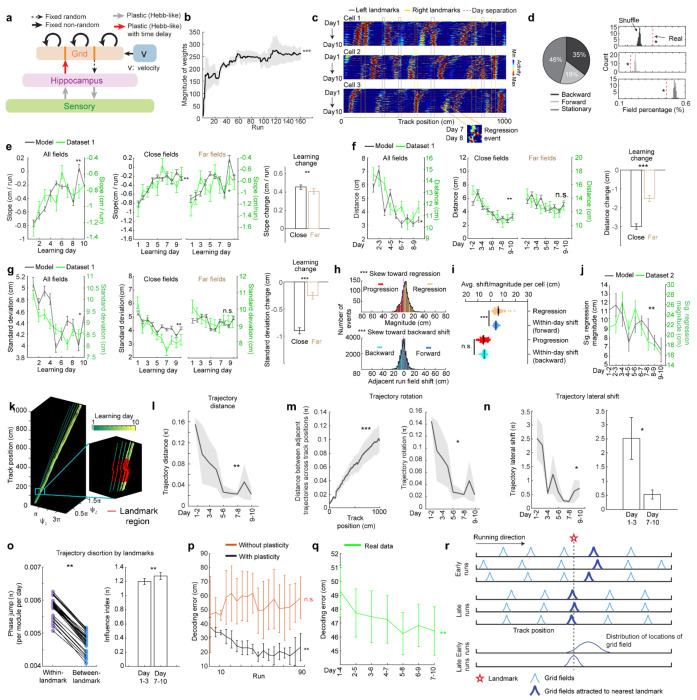
Delayed Hebbian plasticity from hippocampus to grid cells in Vector-HaSH reproduces changes and increased spatial knowledge over map learning **a.** Vector-HaSH model^54^ with the addition of Hebbian plasticity from hippocampus to grid cells. **b.** Magnitude of the weights of the hippocampus-grid synapses across runs. **c.** Cross-day activity patterns of three simulated grid cells, showing shift fields. A field regression event is shown for cell 3 between days 7 and 8. **d.** Fractions of cross-day fields in three shift types. Left: original fraction of detected cross-day fields. Right: comparison of the percentage of three cross-day field types toward the percentage distribution of shuffled fields. **e.** Slope of day fields during learning: for all fields (left), close versus far fields (middle), and learning change from days 1-3 to days 7-10 (right). The calculations were identical to those for real data (Fig. 2h, 2q, and 2t). The curves for real (green) and simulated data (black) are plotted together to demonstrate the consistency, similar in the following plots up to panel **j**. **f.** Similar to **d** but for distances of adjacent daily field centers. **g.** Similar to **d** but for COM standard deviation of run fields. **h.** Distribution of regression and progression events, and within-day adjacent run field shifts in the corresponding directions, across all simulated backward fields. **i.** Comparing the magnitude for regression and progression events toward the within-day adjacent run field shift of the same direction. **j** Magnitude of significant regression events (>70^th^ percentile of run field shifts) across learning. The curves for real (green) and simulated (black) data are plotted together to demonstrate the consistency. **k**. Population activity trajectories of the simulated grid cells across 10 days. Right bottom zoom: all trajectories within the area of a landmark. **l.** Trajectory distance between trajectories in adjacent days in **k**. **m.** Left: trajectory rotations suggested by gradually increasing distances from the beginning to the end of the trajectories across track locations. Right: averaged trajectory rotation across modules between adjacent learning days. **n.** Left: averaged lateral shift between start points of trajectories in adjacent days. Right: comparison between the shifts on day 1-3 and day 7-10 of the learning. **o.** Left: comparison of phase jumps between within- and between-landmark regions. Right: influence index in early (days 1-3) and late learning (days 7-10). **p.** Location decoding error of simulated grid cells from the model with and without plasticity at sensory-to-hippocampus and hippocampus-to-grid synapses across runs. **q.** Location decoding error of real grid cells from dataset 1 across days. **r.** Schematic of noisy grid fields gradually stabilize at landmarks across spatial learning under Hebbian-plasticity based mechanism. *p ≤ 0.05 or the original fraction significantly higher than shuffled fraction in **d** right, **p ≤ 0.01, ***p ≤ 0.001, n.s. p > 0.05. Statistical test results are listed in Table S1. Error bars represent mean ± SEM.

Our model consists of three subcircuits (Fig. 5a): (1) multiple grid cell modules, each modeled as a continuous attractor integrator network^13^, (2) sensory entorhinal cells, whose states are representations of the observed landmarks, and (3) hippocampal cells, which receive inputs from sensory entorhinal cells and grid cells to provide a combined estimate of the animals’ position in the environment. The circuit integrates two types of information about animal position: Grid cells compute position from noisy self-movement velocity estimates, and landmarks send positional information to sensory entorhinal cells (which is represented in the form of high-dimensional feature vectors). Hippocampal cells receive the sensory cell inputs and drive grid cells to influence their population firing phases; then grid cells update their states by integrating their incoming velocity inputs. We assume that the sensory-hippocampus and hippocampus-grid cell synapses undergo slow Hebbian-like synaptic plasticity, and that hippocampal activity is slightly delayed before it influences plasticity in the hippocampus-grid synapses.

We simulated this model on a 1000-cm linear track with eight landmarks, to match experimental conditions in dataset 1. Right or left landmarks activated different subsets of sensory cells (e.g., cue cells in the MEC^42^), and each landmark on a given side induced a different (random and fixed) pattern of sensory activity. The start and end track boundaries were modeled as strong inputs driving both left and right sensory subsets. We used the recorded trajectories of a mouse from dataset 1 which ran 161 runs over 10 days to compute velocity inputs, with the addition of noise.

This model reproduced diverse experimental observations. The hippocampus-to-grid synapses weights gradually increased across runs (Fig. 5b) and drove field shifts over days (Fig. 5c). We observed stationary fields, backward and forward fields (Fig. 5d), with a strong bias towards backward shifts (as in experiments, only the backward fields were statistically significant relative to shuffle Fig. 5d right). The dominance in backward shifts was due to the temporal delay in the heteroassociation between grid and hippocampal cells, with the slope of the backward shift was controlled by the temporal plasticity delay duration (Fig. 5a).

Backward field shifts stabilized over learning as in good performer mice (Fig. 5e–g, left). Stabilization was stronger for fields closer to back landmarks (Fig. 5e–g, middle and right), matching the experimental data. The mechanism of stabilization in the model is a gradual equilibration of the push-pull between delayed plasticity-induced backward field movement and field placement by forward path integration-based phase advance.

Furthermore, adding a slower consolidation timescale to the plasticity process in the same pathway reproduced the partial resetting of learning between days shown in Fig. 3. We assumed that weights continuously update, but if plasticity-inducing experience is interrupted for an extended period, such as the end of a navigation session, they decay to values one consolidation timescale before the interruption. As a result, at the start of each navigation session (“day”), the backward shifting fields exhibited partial position regression (Fig. 5h–j), with decreasing amounts of regression (because there is decreasing drift) over learning.

The population states of grid cells in the model stay on a 2D torus (by construction, as grid modules were low-dimensional continuous attractor networks), so we directly analyzed the evolution of their population phase trajectories over learning. As seen in the mice, the population trajectory exhibits overall shifts (Fig. 5k and l), rotations about the starting point (Fig. 5m), and lateral shifts of the starting points (Fig. 5n), and these dynamics gradually stabilized over learning. Phase trajectories exhibited within-landmark jumps because of the sensory-driven hippocampal inputs (Fig. 5o), and the learned trajectories were increasingly fragmented, with greater landmark influence (larger within-landmark jumps) later in learning (Fig. 4r and s).

**Extended Data Figure 10:**
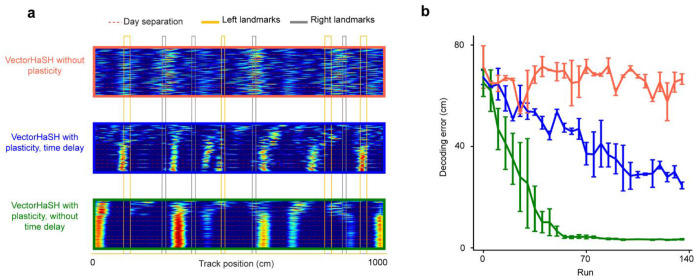
Decoding error of the model in the absence of plasticity or time-delays, related to Figure 5 **a.** Run-by-run cross-day activity of randomly sampled simulated grid cells in each of the three conditions: without any plasticity (orange), with plasticity at sensory-to-hippocampus and time-delayed plasticity hippocampus-to-grid synapses (blue), and with plasticity at sensory-to-hippocampus and hippocampus-to-grid synapses without any time delay (green). **b.** Location decoding error of simulated grid cells from the model in three conditions in **a**.

Finally, with the help of the model, we connected synaptic plasticity-driven field dynamics with spatial learning and function: We examined spatial information by quantifying the accuracy of position decoding from simulated grid cells across learning in our model, finding that the decoding error significantly decreases across learning (Fig. 5p). The decoding error remained higher in an alternate model with no plasticity in the sensory-to-hippocampus and hippocampus-to-grid synapses, and consequently with stationary grid fields (Extended Data Fig.10a and b). When we tested grid cells in mice, we found higher-fidelity spatial representations on later days (i.e., lower decoding error, Fig. 5q). Together with the model, this finding suggests that slow synaptic plasticity in hippocampal-to-grid synapses may be responsible for this increase in spatial fidelity.

Though plasticity in the hippocampus-to-grid cell pathway increases spatial fidelity, the delayed aspect of plasticity (required for backward-shifting fields) is not necessary for accuracy: implementing the plasticity without a delay in the hippocampal activity resulted in improved decoding without backward field shifts (Extended Data Fig.10a and b). Backward shifts are, however, responsible for increasing map fragmentation over learning.

In sum, our model recapitulated many features of experimental data and predicts that Hebbian plasticity from sensory to hippocampal cells and delayed Hebbian plasticity-based from the hippocampus to grid cells is responsible for map learning (Fig. 5r).

### Synaptic change in the indirect hippocampal output to the MEC is associated with spatial learning

We next test our model’s prediction about the plasticity in the hippocampus-to-MEC pathway for spatial learning. We hypothesize that larger hippocampus-grid weights during learning (Fig. 5b) correspond to synaptic potentiation in the superficial layers of the MEC, and should be greater in good performers than in poor performers. We also expect non-saturation of such potentiation in one environment, for good performers to continue learning in new environments.

**Extended Data Figure 11:**
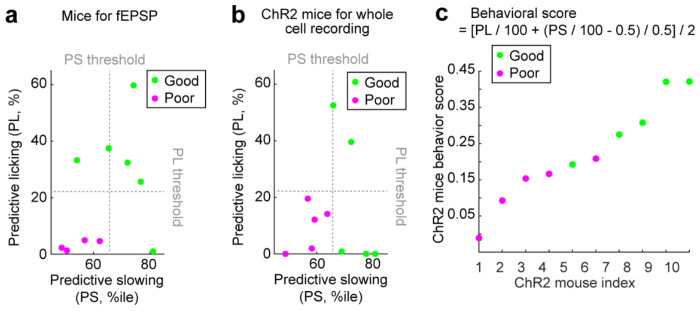
Behavior of mice in the electrophysiology experiment, related to Figure 6 **a.** Predictive licking (PL) and predictive slowing (PS) metric for mice participated in the fEPSP measurement experiment. Averaged PS and PL of last 6 learning days in novel environment were compared to reported PS and PL threshold for good performers (PL threshold: 22.2^th^ percentile , PS threshold: 65.7^th^ percentile)^38^. Mice with both averaged PL and PS below thresholds were classified as poor performers **b.** Same as **a** but for mice participated in the optogenetic stimulation experiment **c**. Behavior score calculated from PL and PS of mice participated in optogenetic stimulation experiment. The score is represented by values from 0 to 1 based on the formula on top.

**Figure 6.**
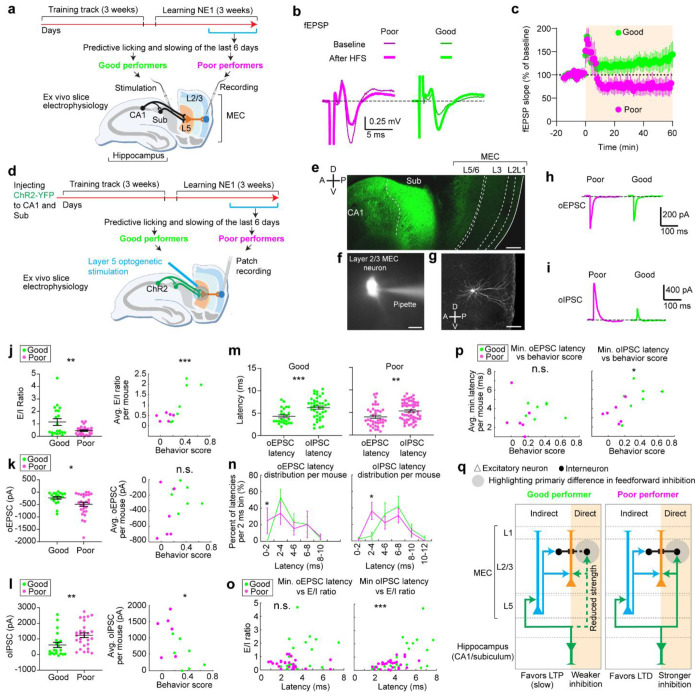
The microcircuits of layer 2 MEC in good and poor performers display different electrophysiological properties **a.** Schematic of the *ex vivo* electrophysiology experiments to recode fEPSPs in MEC layer 2/3 while stimulating hippocampal inputs. **b.** Representative traces of electrically evoked fEPSPs from poor (magenta) and good (green) performers before (thin) and after (thick) high-frequency stimulation (HFS). **c.** Time courses of the normalized fEPSP slope (mean ± SEM) from poor (magenta) and good (green) performers. HFS was given at 0 min. **d.** Schematic of optogenetic stimulation of axonal fibers from CA1/subiculum in layer 5 while performing patch recording from layer 2/3 neurons. **e.** Immunohistology of recoded slice showing Channelrhodopsin-2 expression in CA1 and subiculum. Axonal projections from the hippocampus to MEC layer 5 and layer 2/3 are reflected as dimmer fluorescence. Scale bar: 200 μm. **f.** Example a patched neuron. Scale bar: 15μm **g**. Confocal image of a recorded layer 2/3 neuron. Scale bar: 100μm **h**. Representative traces of optogenetically evoked oEPSCs in MEC layer 2/3 neurons, from a poor (magenta) performer and a good (green) performer. **i**. Similar to **h** but for oIPSC **j.** Left: E/I ratio of individual neurons from good and poor performers. Right: Relationship between behavior score and averaged E/I ratio across neurons for each good and poor performer **k.** Similar to **j** but for oEPSC **l.** Similar to **j** but for oIPSC **m.** Comparison of oEPSC and oIPSC latency distribution for good (left) and poor (right) performers. **n.** Distribution of oEPSC (left) and oIPSC latencies (right) in good and poor performers, binned in every 2 ms. For each mouse, the number of latencies in each 2ms time bin were collected across all cells of the mouse and were normalized by the total number of latencies of the mouse. This produces “percent of latencies in each bin” per mouse. This percent in each 2ms time bin was compared between good and poor performers. **o**. Relationship between averaged minimum oEPSC (left) and oIPSC latency (right) and E/I balance, for individual neurons in good and poor performers. **p**. Relationship between averaged minimum oEPSC (left) and oIPSC latency (right) across neurons of the same mouse versus behavior score of each mouse. **q**. Proposed circuit mechanism underling differential synaptic plasticity in MEC layer 2/3 of good and poor performers. For each performer group, “direct” and “indirect” indicate “direct pathway” and “indirect pathway” from the hippocampus, respectively. The reduced strength of the “direct pathway” in good performers primarily leads to reduced feedforward inhibition, favoring slow LTP induction through in the “indirect pathway”. The stronger inhibition in poor performers favors LTD. *p ≤ 0.05, **p ≤ 0.01, ***p ≤ 0.001, n.s. p > 0.05. Statistical test results are listed in Table S1. Error bars represent mean ± SEM.

We performed *ex vivo* slice electrophysiology to examine synaptic plasticity in the hippocampal projections to the MEC in mice that had learned virtual environments. Mice were trained to navigate VR tracks for two weeks, then learned the same N1 as in dataset 1 for another two weeks (Fig. 6a). Based on reward-predictive behaviors during the last six sessions, mice were classified as good or poor performers using criteria from dataset 1^38^ (Extended Data Fig.11a). All mice were sacrificed within 30-40 minutes after the final behavioral session. *Ex vivo* brain slices containing the MEC and the hippocampus were then prepared to assess synaptic plasticity in MEC layer 2/3 while stimulating the hippocampus^55–57^.

We recorded extracellular field excitatory postsynaptic potentials (fEPSPs) from the MEC layer 2/3 while stimulating axonal fibers from the CA1 and subiculum (Fig. 6a). These fibers primarily convey hippocampal inputs to MEC layer 2/3 via MEC layer 5 (“indirect pathway”)^58–61^, with a recent study also identifying a direct CA1 to MEC layer 2/3 pathway (“direct pathway”)^62^. A 15-minute baseline fEPSP was recorded, followed by high-frequency stimulation (HFS) to induce synaptic plasticity^63^. fEPSPs were monitored for 60 minutes post-HFS. The fEPSP slope changed after HFS (Fig. 6b). Good performers showed an increase in fEPSP slope (125 ± 18% from baseline), indicating long-term potentiation (LTP). Poor performers exhibited a decrease (67 ± 15% from baseline), indicating long-term depression (LTD) (Fig. 6c). These results suggest that hippocampal inputs are more likely to induce synaptic potentiation in MEC layer 2/3 of good performers compared to poor performers.

To explore the mechanisms behind synaptic plasticity differences in good and poor performers, we conducted whole-cell recordings from MEC layer 2/3 neurons while selectively stimulating hippocampal axonal projections using optogenetics. We virally expressed channelrhodopsin-2 variant H134R^64^ in CA1 and subiculum neurons and prepared brain slices from VR-trained mice 4-6 weeks after virus injection (Fig. 6d and e). Mice underwent the same spatial learning task in N1 and were classified as good or poor performers (Extended Data Fig.11b). Brief blue light pulses were applied to the MEC layer 5 to stimulate inputs from CA1 and subiculum. Voltage-clamp recordings from layer 2/3 neurons (Fig. 6f and g) showed excitatory postsynaptic currents (oEPSCs), which were blocked by glutamate receptor blockers (NBQX and CPP) when holding the membrane potential at −60 mV (Fig. 6h). The same optogenetic stimulation also evoked inhibitory postsynaptic currents (oIPSCs), which were blocked by GABA receptor blockers (gabazine) when holding the membrane potential of the neuron at the reversal potential for glutamate receptor currents (0 mV) (Fig. 6i).

We calculated the ratio of oEPSC to oIPSC amplitude (E/I ratio) for each neuron, finding that E/I ratio was higher in neurons of good performers and correlated with learning performance (Fig. 6j, and Extended Data Fig.11c for behavior score calculation). While oEPSC amplitudes showed a small decrease in good performers compared to poor performers, this amplitude did not correlate well with their learning performance (Fig. 6k). In contrast, oIPSC amplitudes were significantly lower in good performers and negatively correlated with learning performance (Fig. 6l). We propose that in MEC layer 2/3 of good performers, there is reduced recruitment of both excitation and inhibition via the hippocampus-to-MEC projection, with a greater reduction in inhibition leading to a higher E/I ratio. This network state likely lowers the threshold for neural activation, increasing the likelihood of synaptic potentiation in good performers.

**Extended Data Figure 12:**
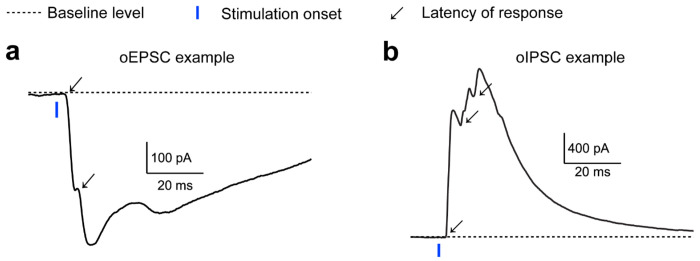
Multiple latencies within oEPSC and oIPSC events, related to Figure 6 **a.** Example oEPSC trace display multiple latency responses. Responses were labeled with arrow. **b.** Similar to a but for example oIPSC trace.

To identify specific synaptic pathways undergoing changes, we analyzed the latency of oEPSC and oIPSC response events relative to the optogenetic stimulation. Each event could have multiple latencies, reflecting multiple presynaptic inputs converging on the same postsynaptic cell (Extended Data Fig.12). After pooling individual latencies, we found that oIPSC latencies were significantly delayed compared to oEPSC latencies in both good and poor performers (Fig. 6m), indicating that inhibition requires the recruitment of more synapses than excitation. This finding is consistent with previous studies showing that while excitatory hippocampal projections reach MEC layer 2/3 through both “direct” and “indirect pathways”, these projections further synapse with interneurons to provide feedforward inhibition^60,62,65^.

We observed that oEPSC latencies in both groups were mostly within 4 ms. However, good performers had a lower percentage of shorter latencies (0-2 ms) (Fig. 6n, left), suggesting that they received less excitation from the “direct pathway”. Meanwhile, oIPSC latencies of poor performers peaked around 2-4 ms and 6-8 ms, but good performers showed reduced latencies around 2-4 ms, and similar latency abundance at 6-8 ms (Fig. 6n, right). This reduced short-latency oIPSC in good performers indicates that they received less feedforward inhibition from the “direct pathway”. Thus, the weakening of the “direct pathway” from the hippocampus to MEC layer 2/3 of good performers could lead to the reduced strengths of oIPSC and oEPSC observed above (Fig. 6k and l).

Additionally, the minimal latency of individual oIPSCs per neuron, which reflected the timing of its fastest inhibitory inputs, positively correlated with the E/I ratio (Fig. 6o). The averaged minimal latencies of oIPSCs in individual mice also positively correlated with their behavioral performances (Fig. 6p). These correlations were not observed for oEPSC latencies. These findings suggest that delayed inhibition in good performers primarily contributes to their higher E/I ratio and better learning performance.

Together, *ex vivo* slice electrophysiology of MEC layer 2 neurons reveals a link between spatial learning and hippocampal-induced synaptic plasticity in the MEC. LTP in MEC layer 2/3 is specifically induced in good performers, potentially supporting the gradual stabilization of their grid cell activity. Our whole cell recording results suggest that this potentiation is facilitated mainly by the weakening of the “direct pathway” from the hippocampus to MEC layer 2/3 (Fig. 6q). While this pathway predominantly drives feedforward inhibition^62^, our results suggest that the reduced inhibition in this pathway increases the E/I ratio, lowering the threshold for neural activation. This circuit feature favors LTP induction when good performers encounter a novel environment, leading to a gradually stabilized grid map. Thus, the LTP is polysynaptic as it is driven primarily by the “indirect pathway”, consistent with the delayed Hebbian plasticity mechanisms proposed by the Vector-HaSH model (Fig. 5). In contrast, stronger recruitment of the direct pathway in poor performers leads to stronger feedforward inhibition, favoring LTD and destabilizing grid cell activity.

## Discussion

Combining longitudinal two-photon calcium imaging and modeling, we uncovered the dynamics of grid fields at the timescale of cognitive map formation during spatial learning. The combined results provide a signature of a delayed Hebbian plasticity mechanism in shaping the map. In a novel environment, grid fields appeared immediately then shifted backward on a run-by-run basis within and across days. During successful but not unsuccessful learning, these fields gradually stabilized at the landmarks preceding them. The shifts partially reset daily, revealing a slower consolidation process requiring online experience. Population dynamics of co-modular grid cells remained on the same 2D toroidal manifold, with their trajectories on the torus slowly rotating, shifting and gradually stabilizing. The model showed that these dynamics can arise from a Hebbian plasticity mechanism in hippocampal-to-grid synapses with slightly delayed hippocampal activity. The time-delayed plasticity from hippocampus to grid cells potentially drives backward shifting grid fields. Overall, in imaging experiments and model, we showed that the plasticity process resulted in a map that became stabilized but more fragmented over time, with improved positional estimation. Finally, our *ex vivo* electrophysiology results demonstrated the association between hippocampus-driven synaptic plasticity in an indirect pathway to MEC with spatial learning performance. The link we find between grid cell stabilization, synaptic plasticity, and successful spatial learning further reinforces the role of grid cells and cognitive maps in memory.

Our findings augment the characterization of short time-scale grid cell dynamics, such as the observation of “one-shot” landmark-driven grid cell responses within a single day^11^, and the very long-term dynamics of hippocampal representational drift over a month^21^. The timescale of a few days is required for animals to fully learn a novel environment as reflected by their behaviors^66–68^, and is also expected from the problem of self-localization and mapping (SLAM), which requires the bootstrapping of positional estimation from landmarks and path integration and the buildup of their associations^25–28^. At this timescale, we find that maps require slow learning and plasticity over extended bouts of experience to properly stabilize and faithfully encode space. This is consistent with the observation of several-day timescale adjustments in the formation of a global grid cell map in landmark-free open fields^29,31,69^. Intriguingly, in a compartmentalized 2D environment where doorways and interior walls serve as local cues, this several-day map acquisition timescale results in the gradual fusion of grid map fragments over time^69^, while in our 1D environment with local landmarks, the circuit appears to retrench on fragmentation, accentuating the discontinuity at each landmark over learning. We hypothesize that this is because of the perceptual and exploratory differences in the two environments – the paths in our environment are unidirectional and the landmarks are strongly localized salient markers with no long-distance cues about the continuity of the space – and that bi-directional exploration should result in map fusion rather than increasing fragmentation.

Indeed, consistent with this hypothesis, backward field shifts are responsible for increasing map fragmentation in our experiments, and in a bidirectionally traversed environment, there is no consistent notion of “backward”. Additionally, hippocampal place fields shift backward under unidirectional 1D track traversal on a similar timescale^24,32–37^. As in our observations on grid cells, the degree of shifting decreases with experience^24^ and there is a between-day partial regression in the shifts^24^. This continuous shifting of place fields over days differs from the transient backward shift of CA1 place fields following burst firing during initial field formation, a hallmark of BTSP^12,70,71^, though a recent study suggests that BTSP could also explain these continuous shifts^72^. Our works suggests an alternate possibility: hippocampal cells in our model are driven by feedforward grid inputs rather than landmark cues over the majority of the track; thus, the properties of grid field shifts should be mirrored in result in corresponding shifts in hippocampal fields (data not shown). Thus our findings raise the possibility that hippocampal shifts on the timescale of days could be grid-cell-driven and due to Hebbian-like learning with a time-delay rather than due to STDP or BTSP^72^.

The time-delay in our proposed plasticity dynamics could stem from synaptic eligibility traces^73^, local recurrent connections in the hippocampus^74^, or information processing delays due to the largely indirect inputs from the hippocampus to layer 2/3 of the MEC^58–61^. While future studies are needed to fully investigate these mechanisms, our *ex vivo* slice physiology supports the latter. We found that CA1/subiculum stimulation induced synaptic potentiation in MEC layer 2/3 of good performers and synaptic depression in poor performers. This difference may be due to the weakening of the “direct pathway” from the hippocampus to MEC layer 2/3, which mainly drives feedforward inhibition^62^. Reduced feedforward inhibition in good performers increases the E/I ratio and lowers the threshold for neural activation, favoring LTP induction via the “indirect pathway” and creating the time-delayed potentiation proposed by the Vector-HaSH model. Conversely, stronger inhibition through the “direct pathway” favors LTD. Additionally, a recent study showed that show that hippocampal stimulation can enhance synaptic responses in MEC layers 2/3 due to heterosynaptic potentiation of cortical inputs^62^. Cortical inputs, which carry multimodal sensory information^75^, might be enhanced in mice with extensive VR experience, further facilitating synaptic potentiation in good performers. The ability to induce LTP in mice that learned an environment indicates that the MEC circuit’s capacity to potentiate is not saturated, allowing continuous learning of new environments.

In summary, our *in vivo* calcium imaging, computational modeling, *ex vivo* electrophysiology, and behavioral analyses demonstrate the link between delayed synaptic plasticity from the hippocampus to the MEC and the stabilization of grid cell activity, revealing an important mechanism underlying spatial map learning.

## Extended Data

## Supplementary Material

1
