# Supplementary material for: Slow synaptic plasticity from the hippocampus underlies gradual mapping and fragmentation of novel spaces by grid cells
Source: bioRxiv. 2025 Nov 24:2025.07.30.667696. Originally published 2025 Jul 30. Preprint. [Version 4] doi: 10.1101/2025.07.30.667696 (PMC12324395; doi:10.1101/2025.07.30.667696)
Supplement: Supplement 1 [file NIHPP2025.07.30.667696v4-supplement-1.pdf]

bioRxiv preprint doi: https://doi.org/10.1101/2025.07.30.667696; this version posted November 24, 2025. The copyright holder for this preprint (which was not certified by peer review) is the author/funder. This article is a US Government work. It is not subject to copyright under 17 USC 105 and is also made available for use under a CC0 license.

Supplementary Materials for

Slow synaptic plasticity from the hippocampus underlies gradual mapping and fragmentation of novel spaces by grid cells

Lujia Chen[1], Ling Liang Dong[2, 7], Hoon Shin[3, 7], Farid Shahid[1, 7], Taylor Joseph Malone[1], Yan Ma[1], Sreerag Othayoth Vasu[3], Nai-Wen Tien[1, 4], Kyle Cekada[1,5], Kevin Jiang Zhang[1,6], Lucy Anderson[3], Sarthak Chandra[2, *], Ila Fiete[2, *], Veronica A. Alvarez[3, *], Yi Gu[1, *]

[*]Co-corresponding authors: Sarthak Chandra: sarthakc@mit.edu; Ila Fiete: fiete@mit.edu; Veronica A. Alvarez: alvarezva@mail.nih.gov; Yi Gu: yi.gu@nih.gov

bioRxiv preprint doi: https://doi.org/10.1101/2025.07.30.667696; this version posted November 24, 2025. The copyright holder for this preprint (which was not certified by peer review) is the author/funder. This article is a US Government work. It is not subject to copyright under 17 USC 105 and is also made available for use under a CC0 license.

Table S1: Statistics of analysis in each figure

Figure 1: (267 neurons of 11 good performer mice, and 267 neurons of 4 poor performer mice)			
j. (Percentile to shuffle distribution)	good backward: 100%		poor backward: 100%
	good forward: 0%		poor forward: 0%
	good stationary: 0%		poor stationary: 0%
k. (Mann-Whitney test, two-tailed)	$p = 1.76\times10^{-4}$		
l. (Mann-Whitney test, two-tailed)	1-day field: p=0.214		
	2-day field: $p=1.898\times10^{-4}$		
	3-day field: p=0.121		
	4-day field: p=0.013		
	5-day field: $p=1.231\times10^{-8}$		
	6-day field: p=0.002		
	7-day field: $p=2.192\times10^{-10}$		
	8-day field: $p=1.247\times10^{-7}$		
	9-day field: p=0.001		
	10-day field: $p=8.710\times10^{-6}$		
Figure 2: (267 neurons of 11 good performer mice, and 267 neurons of 4 poor performer mice)			
b. (curve: Pearson correlation; good vs. poor:repeated measure two-way ANOVA, two-tailed)	Curve (good performer): r=-0.189, p=0.600	Curve (poor performer): r=-0.750, p=0.012	ANOVA(good vs. poor): $p=8.023\times10^{-18}$
c. (curve: Pearson correlation; good vs. poor:repeated measure two-way ANOVA, two-tailed)	Curve (good performer): r=-0.714, p=0.020	Curve (poor performer): r=-0.721, p=0.019	ANOVA (good vs. poor): $p=2.553\times10^{-18}$
d. (curve: Pearson correlation; good vs. poor:repeated measure two-way ANOVA, two-tailed)	Curve (good performer): r=0.829, p=0.006	Curve (poor performer): r=0.619, p=0.07	ANOVA (good vs. poor): p=0.0059
f. (curve: Pearson correlation; good vs. poor:repeated measure two-way ANOVA, two-tailed)	Curve (good performer): r=0.804, p=0.009	Curve (poor performer): r=-0.471, p=0.200	ANOVA (good vs. poor): $p=2.730\times10^{-7}$
h. (curve: Pearson correlation; good vs. poor:repeated measure two-way ANOVA, two-tailed)	Curve (good performer): r=0.685, p=0.029	Curve (poor performer): r=-0.039, p=0.914	ANOVA (good vs. poor): $p=6.879\times10^{-4}$
i. (curve: Pearson correlation; good vs.	Curve (good performer):	Curve (poor performer):	ANOVA (good vs. poor): $p=2.172\times10^{-8}$

bioRxiv preprint doi: https://doi.org/10.1101/2025.07.30.667696; this version posted November 24, 2025. The copyright holder for this preprint (which was not certified by peer review) is the author/funder. This article is a US Government work. It is not subject to copyright under 17 USC 105 and is also made available for use under a CC0 license.

poor:repeated measure two-way ANOVA, two-tailed)	r=0.834, p=0.005	r=-0.566, p=0.112		
j. (curve: Pearson correlation; good vs. poor:repeated measure two-way ANOVA, two-tailed)	Curve (good performer): r=-0.797, p=0.006	Curve (poor performer): r=-0.0215, p=0.551	ANOVA (good vs. poor): $p=1.107\times10^{-13}$	
k. (backward vs. non-backward:repeated measure two-way ANOVA, two-tailed)	ANOVA(Good performer): $p=6.937\times10^{-18}$;		ANOVA(Poor performer): p=0.278	
n .(Pearson correlation)	Good performer: r=-0.952, $p=2.108\times10^{-5}$;		Poor performer: r=-0.683, p=0.0292	
o (top). s (Kolmogorov-Smirnov test between the field center distribution and uniform distribution, two-tailed)	Good performer: p=0.732;		Poor performer: p=0.632	
o (bottom). (Percentile (p) of the original day 7-10 field center fraction toward the fractions of the 1000 simulated normal distribution. p<5% indicates the original field center fraction is below the bottom 5% of simulated fraction, and p>95% indicates the original field center fraction is above the top 5% of simulated fraction. Both cases are regarded as significant (labeled as *)	Good performer		Poor performer	
	-140% to -120%: p=75%			
	-120% to -100%: p=15%		-120% to -100%: p=54%	
	-100% to -80%: p=0.01%		-100% to -80%: p=5%	
	-80% to -60%: p=0%		-80% to -60%: p=0%	
	-60% to -40%: p=0%		-60% to -40%: p=0%	
	-40% to -20%: p=0%		-40% to -20%: p=0%	
	-20% to 0%: p=0%		-20% to 0%: p=0%	
	0% to 20%: p=100%		0% to 20%: p=20%	
	20% to 40%: p=100%		20% to 40%: p=95%	
	40% to 60%: p=100%		40% to 60%: p=100%	
	60% to 80%: p=100%		60% to 80%: p=100%	
	80% to 100%: p=100%		80% to 100%: p=100%	
	100% to 120%: p=0%		100% to 120%: p=0.1%	
q. (curve: Pearson correlation)	Close fields (good): r=0.693, p=0.026	Close fields (poor): r=0.142, p=0.695	Far fields (good): r=0.556, p=0.095	Far fields (poor): r=-0.237, p=0.510
r. (curve: Pearson correlation)	Close fields (good): r=-0.896, p=0.001	Close fields (poor): r=-0.682, p=0.043	Far fields (good): r=-0.731, p=0.025	Far fields (poor): r=-0.191, p=0.622

bioRxiv preprint doi: https://doi.org/10.1101/2025.07.30.667696; this version posted November 24, 2025. The copyright holder for this preprint (which was not certified by peer review) is the author/funder. This article is a US Government work. It is not subject to copyright under 17 USC 105 and is also made available for use under a CC0 license.

s. (curve: Pearson correlation)	Close fields (good): r=-0.810, p=0.004	Close fields (poor): r=-0.234, p=0.516	Far fields (good): r=-0.653, p=0.041	Far fields (poor): r=-0.053, p=0.885
t.(Mann-Whitney U test, two-tailed)	Good close vs. far : p=0.004		Poor close vs. far : p=0.507	
u. (Mann-Whitney U test, two-tailed)	Good close vs. far : $p=1.859\times10^{-7}$		Poor close vs. far : p=0.0004	
v. (Mann-Whitney U test, two-tailed)	Good close vs. far : $p=7.61\times10^{-7}$		Poor close vs. far : p=0.302	
Figure 3. (267 neurons of 11 good performer mice (dataset 1))				
d. (one-sample t-test, two-tailed)	regression/progression: Average shift=2.500 cm forward, $p=1.42\times10^{-14}$		Forward/backward within-day shift: Average shift=0.874cm backward, $p=1.77\times10^{-65}$	
e. (Mann-Whitney U test, two-tailed)	regression vs. progression: $p=1.026\times10^{-4}$ (regression: 4.642; progression :3.291)			
f. (Mann-Whitney U test, two-tailed)	regression vs. forward within-day shift: $p=7.555\times10^{-10}$ (Avg.regression shift: 10.984cm; Avg. forward within-day shift: 7.956cm)		progression vs. backward within-day shift: p=0.4824 (Avg. progression shift: 9.866cm; Avg. backward within-day shift: 8.488cm)	
g. (Pearson correlation)	p=0.0461			
Figure 4. (a,b,c: 114 cell from 11 co-modular grid cell populations) (j,k,m,o,r,s: 75 neurons from 5 co-modular grid cell populations (dataset 1))				
a (Middle):(Real vs. shuffle: repeated measure two-way ANOVA, two-tailed)	p=0			
a(Right): (Mann-Whitney U test, two-tailed)	p=0			
c.(Pearson correlation)	r=-0.8238, p=0.0034			
j. (Pearson correlation)	r=-0.678, p=0.045			
k. (Pearson correlation)	r=0.933, p=0			
m. (Pearson correlation)	r=0.7029, p=0.0347			
o (left) (Pearson correlation)	r=-0.5048, p=0.1657			
o (right), (Mann-Whitney U test, two-tailed)	p=0.0159			
r. (Wilcoxon signed rank test, two side)	p=0.0058			
s. (Mann-Whitney U test, two-tailed)	p=0.0032			

bioRxiv preprint doi: https://doi.org/10.1101/2025.07.30.667696; this version posted November 24, 2025. The copyright holder for this preprint (which was not certified by peer review) is the author/funder. This article is a US Government work. It is not subject to copyright under 17 USC 105 and is also made available for use under a CC0 license.

Figure 5. (a-j: 120 simulated grid cells)(l-o: 4 simulation runs) (p: 11 field of views)		
b. (Pearson correlation)	r=0.7785, $p=5.613\times10^{-34}$	
d. (percentile to shuffle)	backward: 100%, forward: 3%, stationary: 2%	
e (left). (black curve: Pearson correlation)	model: r=-0.765, p=0.0099	
f (left). (black curve: Pearson correlation)	model: r=-0.786, p=0.012	
g (left). (black curve: Pearson correlation)	model r=-0.646, p=0.043	
e (middle). (black curve: Pearson correlation)	Close fields (model): r=0.822, p=0.004	Far fields (model): r=0.669, p=0.034
f (middle). (black curve: Pearson correlation)	Close fields (model): r=-0.816, p=0.007	Far fields (model): r=-0.564, p=0.114
g (middle). (black curve: Pearson correlation)	Close fields (model): r=-0.792, p=0.006	Far fields (good): r=-0.254, p=0.479
e (right). (Mann-Whitney U test, two-tailed)	Model close vs. far : p=0.003	
f (right). (Mann-Whitney U test, two-tailed)	Model close vs. far : $p=5.226\times10^{-18}$	
g (right). (Mann-Whitney U test, two-tailed)	Model close vs. far : $p=1.001\times10^{-14}$	
h. (one-sample t-test, two-tailed)	Regression/progression: Average shift=2.556 cm forward, $p=2.04\times10^{-22}$	Forward/backward within-day shift: Average shift=0.336 cm backward, $p=4.96\times10^{-16}$
i.(Mann-Whitney U test, two-tailed)	Regression vs. forward within-day shift: $p=6.83\times10^{-6}$ (Avg.regression shift: 6.427cm; Avg. forward within-day shift: 4.530cm)	Progression vs. backward within-day shift: p=0.128 (Avg. progression shift: 5.425cm; Avg. backward within-day shift: 4.990cm)
j. (Pearson correlation)	r=-0.808, p=0.0084	
l. (Pearson correlation)	r=-0.912, p=0.006	
m (left). (Pearson correlation)	r=0.994, p=0	
m (right). (Pearson correlation)	r=-0.866, p=0.001	
n (left). (Pearson correlation)	r=-0.796, p=0.010	
n (right). (Mann-Whitney U test, two-tailed)	p=0.0286	
o (left). (Mann-Whitney U test, two-tailed)	$p=3.569\times10^{-8}$	

bioRxiv preprint doi: https://doi.org/10.1101/2025.07.30.667696; this version posted November 24, 2025. The copyright holder for this preprint (which was not certified by peer review) is the author/funder. This article is a US Government work. It is not subject to copyright under 17 USC 105 and is also made available for use under a CC0 license.

o(right). (Mann-Whitney U test, two-tailed)	p=0.042	
p. (Pearson correlation)	With plasticity: r=-0.8188, p=3.2519×10^{-5}	Without plasticity: r=0.1464, p=0.5622
q.(Pearson correlation)	r=-0.8760, p=0.0097	
Figure 6. (a-c: 92 cells from 10 mice) (d-p: 63 cells from 10 mice)		
j (left: Student's t-test; right: pearson correlation)	left: p=0.0051	right: r=0.8136, p=0.0007
k (left: Student's t-test; right: pearson correlation)	left:p=0.0211	right: r=0.3324, p=0.2670
l (left: Student's t-test; right: pearson correlation)	left:p=0.0145	right: r=-0.5929, p=0.0327
m (Mann-Whitney test)	good: p=0.0007	poor: p=0.0034
n (Mann-Whitney test)	oEPSC latency time bins	oIPSC latency time bins
	0-2ms: p=0.0420	0-2ms: p=1.000
	2-4ms: p=0.2902	2-4ms: p=0.0210
	4-6ms: p=0.6562	4-6ms: p=0.8194
	6-8ms: p=0.6562	6-8ms: p=0.5466
	8-10ms: p=0.8741	8-10ms: p=0.8077
		10-12ms: p=0.5385
o. (Pearson correlation)	oEPSC latency- behavior score: r=0.0429,p=0.8893	oIPSC latency- behavior score: r=0.6146, p=0.0254
p.(Pearson correlation)	oEPSC latency-E/I ratio: r=0.2512,p=0.0921	oIPSC latency-E/I ratio: r=0.5903,p=0.00001
Fig.S2. (267 neurons of 11 good performer mice, and 267 neurons of 4 poor performer mice)		
b. (trend: Pearson correlation)	good: r=-0.0333, p0.8183	poor: r=-0.0383, p0.7916
Fig.S4. (267 neurons of 11 good performer mice, and 267 neurons of 4 poor performer mice)		
a . (Mann-Whitney U test, two-tailed)	1-day field: p=9.338×10^{-4}	
	2-day field: p=4.529×10^{-5}	
	3-day field: p=0.329	
	4-day field: p=0.021	
	5-day field: p=0.001	
	6-day field: p=0.007	
	7-day field: p=0.109	
	8-day field: p=0.082	
	9-day field: p=4.194×10^{-5}	
	10-day field: p=0.001	

bioRxiv preprint doi: https://doi.org/10.1101/2025.07.30.667696; this version posted November 24, 2025. The copyright holder for this preprint (which was not certified by peer review) is the author/funder. This article is a US Government work. It is not subject to copyright under 17 USC 105 and is also made available for use under a CC0 license.

c. (curve: Pearson correlation; good vs. poor:repeated measure two-way ANOVA, two-tailed)	Curve (good performer): r=-0.024, p=0.948	Curve (poor performer): r=-0.503, p=0.139	ANOVA(good vs. poor): p=7.532×10-6	
d. (curve: Pearson correlation; good vs. poor:repeated measure two-way ANOVA, two-tailed)	Curve (good performer): r=-0.663, p=0.037	Curve (poor performer): r=-0.653, p=0.041	ANOVA (good vs. poor): p=0.128	
e. (curve: Pearson correlation; good vs. poor:repeated measure two-way ANOVA, two-tailed)	Curve (good performer): r=0.811, p=0.008	Curve (poor performer): r=-0.650, p=0.058	ANOVA (good vs. poor): p=0.477	
f. (curve: Pearson correlation; good vs. poor:repeated measure two-way ANOVA, two-tailed)	Curve (good performer): r=0.644, p=0.061	Curve (poor performer): r=-0.916, p=0.0005	ANOVA (good vs. poor): p=0.033	
g. (curve: Pearson correlation; good vs. poor:repeated measure two-way ANOVA, two-tailed)	Curve (good performer): r=-0.434, p=0.210	Curve (poor performer): r=-0.300, p=0.400	ANOVA (good vs. poor): p=0.0023	
h. (curve: Pearson correlation; good vs. poor:repeated measure two-way ANOVA, two-tailed)	Curve (good performer): r=-0.878, p=0.002	Curve (poor performer): r=-0.529, p=0.143	ANOVA (good vs. poor): p=0.0035	
i. (curve: Pearson correlation; good vs. poor:repeated measure two-way ANOVA, two-tailed)	Curve (good performer): r=-0.682, p=0.030	Curve (poor performer): r=-0.503, p=0.139	ANOVA (good vs. poor): p=0.278	
Fig.S5: (267 neurons of 11 good performer mice, and 267 neurons of 4 poor performer mice)				
a. (curve: Pearson correlation)	Close fields (good): r=-0.316, p=0.373	Curve (poor performer): r=-0.009, p=0.980	Far fields (good): r=-0.416, p=0.232	Far fields (poor): r=-0.365,p=0.299
b. (curve: Pearson correlation)	Close fields (good): r=-917, p=0.0005	Close fields (poor): r=--0.298, p=0.436	Far fields (good): r=-0.814, p=0.008	Far fields (poor): r=-0.034, p=0.931
c. (curve: Pearson correlation)	Close fields (good): r=-0.460, p=0.181	Close fields (poor): r=-0.430,p=0.215	Far fields (good): r=-0.699, p=0.025	Far fields (poor): r=-0.211, p=0.558
d. (Mann-Whitney U test, two-tailed)	Good close vs. far : p=0.722		Poor close vs. far : p=0.258	

bioRxiv preprint doi: https://doi.org/10.1101/2025.07.30.667696; this version posted November 24, 2025. The copyright holder for this preprint (which was not certified by peer review) is the author/funder. This article is a US Government work. It is not subject to copyright under 17 USC 105 and is also made available for use under a CC0 license.

e. (Mann-Whitney U test, two-tailed)	Good close vs. far : p=0.0055		Poor close vs. far : p=0.120	
f. (Mann-Whitney U test, two-tailed)	Good close vs. far : p=0.359		Poor close vs. far : p=0.060	
Fig.S6: (267 neurons of 11 good performer mice)				
b. (Mann-Whitney U test, two-tailed)	regression vs. forward within-day shift (Good): p=0.019 (Avg.regression shift: 9.503 cm; Avg. forward within-day shift: 7.798 cm)	progression vs. backward within-day shift (Good): p=8.297×10-5 (Avg. progression shift: 10.834 cm; Avg. backward within-day shift: 8.226 cm)	regression vs. forward within-day shift (Poor): p=0.167 (Avg.regression shift: 11.835 cm; Avg. forward within-day shift: 10.309 cm)	progressionvs. backward within-day shift (Poor): p=0.292 (Avg. progression shift: 12.880 cm; Avg. backward within-day shift: 10.497cm)
c. (Mann-Whitney U test, two-tailed)	regression (dataset 2) vsregression (dataset1): p=0.0097 (Avg.regression (dataset2): 10.98cm, Avg.regression(dataset 1): 9.503cm)		Forward within-day shift (dataset 2) vs Forward within-day shift (dataset1): p=0.1396 (Avg. forward within-day shift (dataset2): 7.956cm, Avg. forward within-day shift (dataset 1): 7.798cm)	
e. (Pearson correlation)	r=-0.8032, p=0.0091			
Fig.S7: (428 neurons of 8 good performer mice)				
e. (Pearson correlation)	r=0.855, p=0.002			
f. (Pearson correlation)	r=-0.839, p=0.005			
g. (Pearson correlation)	r=-0.892, p=0.0005			
i. (curve: Pearson correlation; bar plot: Mann-Whitney U test, two side)	Close fields (Slope curve): r=0.711, p=0.021	Far fields 1 (Slope curve): r=0.904, p=0.0003	Far fields 2 (Slope curve): r=0.635, p=0.048	
	Close fields (Field center distance curve): r=-	Far Field 1 (Field center distance curve): r=-	Far fields 2 (Field center distance curve): r=-0.789, p=0.012	

bioRxiv preprint doi: https://doi.org/10.1101/2025.07.30.667696; this version posted November 24, 2025. The copyright holder for this preprint (which was not certified by peer review) is the author/funder. This article is a US Government work. It is not subject to copyright under 17 USC 105 and is also made available for use under a CC0 license.

	0.722, p=0.028	0.868, p=0.002	
	Close fields (Standard deviation curve): r=-0.907, p=0.00003	Far Field 1 (Standard deviation curve): r=-0.890, p=0.0006	Far fields 2 (Standard deviation curve): r=-0.691, p=0.027
	Close field vs. far field 1 (slope change) : p=0.267		Close field vs. far field 2 (slope change) : p=0.034
	Close field vs. far field 1 (slope change) : p=0.004		Close field vs. far field 2 (slope change) : p=0.635
	Close field vs. far field 1 (slope change) : $p=3.875\times10^{-8}$		Close field vs. far field 2 (slope change) : $p=2.213\times10^{-14}$
