# Supplementary material for: Slow synaptic plasticity from the hippocampus underlies gradual mapping and fragmentation of novel spaces by grid cells
Source: bioRxiv. 2025 Jul 30:2025.07.30.667696. Preprint. [Version 1] doi: 10.1101/2025.07.30.667696 (PMC12324395; doi:10.1101/2025.07.30.667696)
Supplement: Supplementary file 1 [file NIHPP2025.07.30.667696v1-supplement-1.pdf]

bioRxiv preprint doi: https://doi.org/10.1101/2025.07.30.667696; this version posted July 30, 2025. The copyright holder for this preprint (which was not certified by peer review) is the author/funder. This article is a US Government work. It is not subject to copyright under 17 USC 105 and is also made available for use under a CC0 license.

Supplementary information for

Slow synaptic plasticity from the hippocampus underlies gradual mapping and fragmentation of novel spaces by grid cells

Lujia Chen[1], Ling Liang Dong[2, 5], Hoon Shin[3, 5], Farid Shahid[1, 5], Taylor Malone[1], Yan Ma[1], Sreerag Othayoth Vasu[3], Nai-Wen Tien[1, 4], Kyle Cekada[1], Lucy Anderson[3], Sarthak Chandra[2, *], Ila Fiete[2, *], Veronica A. Alvarez[3, *], Yi Gu[1, *]

[*]Co-corresponding authors: Sarthak Chandra: sarthakc@mit.edu; Ila Fiete: fiete@mit.edu; Veronica A. Alvarez: alvarezva@mail.nih.gov; Yi Gu: yi.gu@nih.gov

bioRxiv preprint doi: https://doi.org/10.1101/2025.07.30.667696; this version posted July 30, 2025. The copyright holder for this preprint (which was not certified by peer review) is the author/funder. This article is a US Government work. It is not subject to copyright under 17 USC 105 and is also made available for use under a CC0 license.

Table S1: Statistics of analysis in each figure

Figure 1: (267 neurons of 11 good performer mice, and 267 neurons of 4 poor performer mice)		
j. (Percentile to shuffle distribution)	good backward: 100%	poor backward: 100%
	good forward: 0%	poor forward: 0%
	good stationary: 0%	poor stationary: 0%
k. (Mann-Whitney test, two-tailed)	p = 1.76×10^{-4}	
l. (Mann-Whitney test, two-tailed)	1-day field: p=0.214	
	2-day field: p=1.898×10^{-4}	
	3-day field: p=0.121	
	4-day field: p=0.013	
	5-day field: p=1.231×10^{-8}	
	6-day field: p=0.002	
	7-day field: p=2.192×10^{-10}	
	8-day field: p=1.247×10^{-7}	
	9-day field: p=0.001	
	10-day field: p=8.710×10^{-6}	

Figure 2: (267 neurons of 11 good performer mice, and 267 neurons of 4 poor performer mice)			
b. (curve: Pearson correlation; good vs. poor:repeated measure two-way ANOVA, two-tailed)	Curve (good performer): r=-0.189, p=0.600	Curve (poor performer): r=-0.750, p=0.012	ANOVA(good vs. poor): p=8.023×10^{-18}
c. (curve: Pearson correlation; good vs. poor:repeated measure two-way ANOVA, two-tailed)	Curve (good performer): r=-0.714, p=0.020	Curve (poor performer): r=-0.721, p=0.019	ANOVA (good vs. poor): p=2.553×10^{-18}
d. (curve: Pearson correlation; good vs. poor:repeated measure two-way ANOVA, two-tailed)	Curve (good performer): r=0.829, p=0.006	Curve (poor performer): r=0.619, p=0.07	ANOVA (good vs. poor): p=0.0059
f. (curve: Pearson correlation; good vs. poor:repeated measure two-way ANOVA, two-tailed)	Curve (good performer): r=0.804, p=0.009	Curve (poor performer): r=-0.471, p=0.200	ANOVA (good vs. poor): p=2.730×10^{-7}
h. (curve: Pearson correlation; good vs. poor:repeated measure two-way ANOVA, two-tailed)	Curve (good performer): r=0.685, p=0.029	Curve (poor performer): r=-0.039, p=0.914	ANOVA (good vs. poor): p=6.879×10^{-4}
i. (curve: Pearson correlation; good vs. poor:repeated measure two-way ANOVA, two-tailed)	Curve (good performer): r=0.834, p=0.005	Curve (poor performer): r=-0.566, p=0.112	ANOVA (good vs. poor): p=2.172×10^{-8}
j. (curve: Pearson correlation; good vs. poor:repeated measure two-way ANOVA, two-tailed)	Curve (good performer): r=-0.797, p=0.006	Curve (poor performer): r=-0.0215, p=0.551	ANOVA (good vs. poor): p=1.107×10^{-13}

bioRxiv preprint doi: https://doi.org/10.1101/2025.07.30.667696; this version posted July 30, 2025. The copyright holder for this preprint (which was not certified by peer review) is the author/funder. This article is a US Government work. It is not subject to copyright under 17 USC 105 and is also made available for use under a CC0 license.

k. (backward vs. non-backward:repeated measure two-way ANOVA, two-tailed)	ANOVA(Good performer): $p=6.937\times10^{-18}$;		ANOVA(Poor performer): p=0.278	
c .(Pearson correlation)	Good performer: r=-0.952, $p=2.108\times10^{-5}$;		Poor performer: r=-0.683, p=0.0292	
d (top). s (Kolmogorov-Smirnov test between the field center distribution and uniform distribution, two-tailed)	Good performer: p=0.732;		Poor performer: p=0.632	
d (bottom). (Percentile (p) of the original day 7-10 field center fraction toward the fractions of the 1000 simulated normal distribution. p<5% indicates the original field center fraction is below the bottom 5% of simulated fraction, and p>95% indicates the original field center fraction is above the top 5% of simulated fraction. Both cases are regarded as significant (labeled as *)	Good performer		Poor performer	
	-140% to -120%: p=75%			
	-120% to -100%: p=15%		-120% to -100%: p=54%	
	-100% to -80%: p=0.01%		-100% to -80%: p=5%	
	-80% to -60%: p=0%		-80% to -60%: p=0%	
	-60% to -40%: p=0%		-60% to -40%: p=0%	
	-40% to -20%: p=0%		-40% to -20%: p=0%	
	-20% to 0%: p=0%		-20% to 0%: p=0%	
	0% to 20%: p=100%		0% to 20%: p=20%	
	20% to 40%: p=100%		20% to 40%: p=95%	
	40% to 60%: p=100%		40% to 60%: p=100%	
	60% to 80%: p=100%		60% to 80%: p=100%	
	80% to 100%: p=100%		80% to 100%: p=100%	
	100% to 120%: p=0%		100% to 120%: p=0.1%	
f. (curve: Pearson correlation)	Close fields (good): r=0.693, p=0.026	Close fields (poor): r=0.142, p=0.695	Far fields (good): r=0.556, p=0.095	Far fields (poor): r=-0.237, p=0.510
g. (curve: Pearson correlation)	Close fields (good): r=-0.896, p=0.001	Close fields (poor): r=-0.682, p=0.043	Far fields (good): r=-0.731, p=0.025	Far fields (poor): r=-0.191, p=0.622
h. (curve: Pearson correlation)	Close fields (good): r=-0.810, p=0.004	Close fields (poor): r=-0.234, p=0.516	Far fields (good): r=-0.653, p=0.041	Far fields (poor): r=-0.053, p=0.885
i.(Mann-Whitney U test, two-tailed)	Good close vs. far : p=0.004		Poor close vs. far : p=0.507	
j. (Mann-Whitney U test, two-tailed)	Good close vs. far : $p=1.859\times10^{-7}$		Poor close vs. far : p=0.0004	
k. (Mann-Whitney U test, two-tailed)	Good close vs. far : $p=7.61\times10^{-7}$		Poor close vs. far : p=0.302	
n .(Pearson correlation)	Good performer: r=-0.952, $p=2.108\times10^{-5}$;		Poor performer: r=-0.683, p=0.0292	
o (top). s (Kolmogorov-Smirnov test between the field center distribution and uniform distribution, two-tailed)	Good performer: p=0.732;		Poor performer: p=0.632	
o (bottom). (Percentile (p) of the original day 7-10 field center fraction toward the fractions of the 1000 simulated normal distribution. p<5% indicates the original field center fraction is below the bottom 5% of simulated fraction, and p>95% indicates the original field center fraction is above the top 5% of simulated fraction. Both cases are regarded as significant (labeled as *)	Good performer		Poor performer	
	-140% to -120%: p=75%			
	-120% to -100%: p=15%		-120% to -100%: p=54%	
	-100% to -80%: p=0.01%		-100% to -80%: p=5%	
	-80% to -60%: p=0%		-80% to -60%: p=0%	
	-60% to -40%: p=0%		-60% to -40%: p=0%	
	-40% to -20%: p=0%		-40% to -20%: p=0%	
	-20% to 0%: p=0%		-20% to 0%: p=0%	
	0% to 20%: p=100%		0% to 20%: p=20%	

bioRxiv preprint doi: https://doi.org/10.1101/2025.07.30.667696; this version posted July 30, 2025. The copyright holder for this preprint (which was not certified by peer review) is the author/funder. This article is a US Government work. It is not subject to copyright under 17 USC 105 and is also made available for use under a CC0 license.

	20% to 40%: p=100%		20% to 40%: p=95%	
	40% to 60%: p=100%		40% to 60%: p=100%	
	60% to 80%: p=100%		60% to 80%: p=100%	
	80% to 100%: p=100%		80% to 100%: p=100%	
	100% to 120%: p=0%		100% to 120%: p=0.1%	
q. (curve: Pearson correlation)	Close fields (good): r=0.693, p=0.026	Close fields (poor): r=0.142, p=0.695	Far fields (good): r=0.556, p=0.095	Far fields (poor): r=-0.237, p=0.510
r. (curve: Pearson correlation)	Close fields (good): r=-0.896, p=0.001	Close fields (poor): r=-0.682, p=0.043	Far fields (good): r=-0.731, p=0.025	Far fields (poor): r=-0.191, p=0.622
s. (curve: Pearson correlation)	Close fields (good): r=-0.810, p=0.004	Close fields (poor): r=-0.234, p=0.516	Far fields (good): r=-0.653, p=0.041	Far fields (poor): r=-0.053, p=0.885
t.(Mann-Whitney U test, two-tailed)	Good close vs. far : p=0.004		Poor close vs. far : p=0.507	
u. (Mann-Whitney U test, two-tailed)	Good close vs. far : p=1.859×10^{-7}		Poor close vs. far : p=0.0004	
v. (Mann-Whitney U test, two-tailed)	Good close vs. far : p=7.61×10^{-7}		Poor close vs. far : p=0.302	
Figure 3. (267 neurons of 11 good performer mice (dataset 1))				
d. (one-sample t-test, two-tailed)	regression/progression: Average shift=2.500 cm forward, p=1.42×10^{-14}		Forward/backward within-day shift: Average shift=0.874cm backward, p=1.77×10^{-65}	
e. (Mann-Whitney U test, two-tailed)	regression vs. progression: p=1.026×10^{-4} (regression: 4.642; progression :3.291)			
f. (Mann-Whitney U test, two-tailed)	regression vs. forward within-day shift: p=7.555×10-10 (Avg.regression shift: 10.984cm; Avg. forward within-day shift: 7.956cm)		progression vs. backward within-day shift: p=0.4824 (Avg. progression shift: 9.866cm; Avg. backward within-day shift: 8.488cm)	
g. (Pearson correlation)	p=0.0461			
Figure 4. (a,b,c: 114 cell from 11 co-modular grid cell populations) (j,k,m,o,r,s: 75 neurons from 5 co-modular grid cell populations (dataset 1))				
a (Middle):(Real vs. shuffle: repeated measure two-way ANOVA, two-tailed)	p=0			
a(Right): (Mann-Whitney U test, two-tailed)	p=0			
b.(Pearson correlation)	r=0.7785, p=5.6134×10^{-34}			
c.(Pearson correlation)	r=-0.8238, p=0.0034			
j. (Pearson correlation)	r=-0.678, p=0.045			
k. (Pearson correlation)	r=0.933, p=0			
m. (Pearson correlation)	r=0.7029, p=0.0347			
o (left) (Pearson correlation)	r=-0.5048, p=0.1657			
o (right), (Mann-Whitney U test, two-tailed)	p=0.0362			
r. (Wilcoxon signed rank test, two side)	p=0.0058			
s. (Mann-Whitney U test, two-tailed)	p=0.0032			
Figure 5. (a-i: 120 simulated grid cells) (p: 11 field of views)				
c. (percentage to shuffle)	backward: 100%			
	forward: 3%			
	stationary: 2%			
d (left). (black curve: Pearson correlation)	model: r=-0.765, p=0.0099			
e (left). (black curve: Pearson correlation)	model: r=-0.786, p=0.012			

bioRxiv preprint doi: https://doi.org/10.1101/2025.07.30.667696; this version posted July 30, 2025. The copyright holder for this preprint (which was not certified by peer review) is the author/funder. This article is a US Government work. It is not subject to copyright under 17 USC 105 and is also made available for use under a CC0 license.

f (left). (black curve: Pearson correlation)	model r=-0.646, p=0.043	
d (middle). (black curve: Pearson correlation)	Close fields (model): r=0.822, p=0.004	Far fields (model): r=0.669, p=0.034
e (middle). (black curve: Pearson correlation)	Close fields (model): r=-0.816, p=0.007	Far fields (model): r=-0.564, p=0.114
f (middle). (black curve: Pearson correlation)	Close fields (model): r=-0.792, p=0.006	Far fields (good): r=-0.254, p=0.479
d (right). (Mann-Whitney U test, two-tailed)	Model close vs. far : p=0.003	
e (right). (Mann-Whitney U test, two-tailed)	Model close vs. far : $p=5.226\times10^{-18}$	
f (right). (Mann-Whitney U test, two-tailed)	Model close vs. far : $p=1.001\times10^{-14}$	
g. (one-sample t-test, two-tailed)	Regression/progression: Average shift=2.556 cm forward, $p=2.04\times10^{-22}$	Forward/backward within-day shift: Average shift=0.336 cm backward, $p=4.96\times10^{-16}$
h.(Mann-Whitney U test, two-tailed)	Regression vs. forward within-day shift: p=6.83×10-6 (Avg.regression shift: 6.427cm; Avg. forward within-day shift: 4.530cm)	Progression vs. backward within-day shift: p=0.128 (Avg. progression shift: 5.425cm; Avg. backward within-day shift: 4.990cm)
i. (Pearson correlation)	r=-0.808, p=0.0084	
k. (Pearson correlation)	r=-0.857, p=0.003	
l (left). (Pearson correlation)	r=0.993, p=0	
l (right). (Pearson correlation)	r=-0.866, p=0.003	
m (left). (Pearson correlation)	r=-0.742, p=0.022	
m (right). (Mann-Whitney U test, two-tailed)	p=0.0364	
n (left). (Mann-Whitney U test, two-tailed)	p=4.504×10-11	
n(right). (Mann-Whitney U test, two-tailed)	p=0.0036	
o. (Pearson correlation)	With plasticity: r=-0.8188, $p=3.2519\times10^{-5}$	Without plasticity: r=0.1464, p=0.5622
p.(Pearson correlation)	r=-0.8760, p=0.0097	
Figure 6. (a-c: 92 cells from 10 mice) (d-p: 63 cells from 10 mice)		
j (left: Student's t-test; right: pearson correlation)	left: p=0.0051	right: r=0.8136, p=0.0007
k (left: Student's t-test; right: pearson correlation)	left:p=0.0211	right: r=0.3324, p=0.2670
l (left: Student's t-test; right: pearson correlation)	left:p=0.0145	right: r=-0.5929, p=0.0327
m (Mann-Whitney test)	good: p=0.0007	poor: p=0.0034
n (Mann-Whitney test)	oEPSC latency time bins	oIPSC latency time bins
	0-2ms: p=0.0420	0-2ms: p=1.000
	2-4ms: p=0.2902	2-4ms: p=0.0210
	4-6ms: p=0.6562	4-6ms: p=0.8194
	6-8ms: p=0.6562	6-8ms: p=0.5466
	8-10ms: p=0.8741	8-10ms: p=0.8077
		10-12ms: p=0.5385
o. (Pearson correlation)	oEPSC latency- behavior score: r=0.0429, p=0.8893	oIPSC latency- behavior score: r=0.6146, p=0.0254
p.(Pearson correlation)	oEPSC latency-E/I ratio: r=0.2512,p=0.0921	oIPSC latency-E/I ratio: r=0.5903,p=0.00001
Extended data figure 2. (267 neurons of 11 good performer mice, and 267 neurons of 4 poor performer mice)		

bioRxiv preprint doi: https://doi.org/10.1101/2025.07.30.667696; this version posted July 30, 2025. The copyright holder for this preprint (which was not certified by peer review) is the author/funder. This article is a US Government work. It is not subject to copyright under 17 USC 105 and is also made available for use under a CC0 license.

b. (trend: Pearson correlation)	good: r=-0.0333, p0.8183		poor: r=-0.0383, p0.7916	
Extended data figure 4. (267 neurons of 11 good performer mice, and 267 neurons of 4 poor performer mice)				
a . (Mann-Whitney U test, two-tailed)	1-day field: $p=9.338\times10^{-4}$			
	2-day field: $p=4.529\times10^{-5}$			
	3-day field: p=0.329			
	4-day field: p=0.021			
	5-day field: p=0.001			
	6-day field: p=0.007			
	7-day field: p=0.109			
	8-day field: p=0.082			
	9-day field: $p=4.194\times10^{-5}$			
	10-day field: p=0.001			
c. (curve: Pearson correlation; good vs. poor:repeated measure two-way ANOVA, two-tailed)	Curve (good performer): r=-0.024, p=0.948	Curve (poor performer): r=-0.503, p=0.139	ANOVA(good vs. poor): $p=7.532\times10^{-6}$	
d. (curve: Pearson correlation; good vs. poor:repeated measure two-way ANOVA, two-tailed)	Curve (good performer): r=-0.663, p=0.037	Curve (poor performer): r=-0.653, p=0.041	ANOVA (good vs. poor): p=0.128	
e. (curve: Pearson correlation; good vs. poor:repeated measure two-way ANOVA, two-tailed)	Curve (good performer): r=0.811, p=0.008	Curve (poor performer): r=-0.650, p=0.058	ANOVA (good vs. poor): p=0.477	
f. (curve: Pearson correlation; good vs. poor:repeated measure two-way ANOVA, two-tailed)	Curve (good performer): r=0.644, p=0.061	Curve (poor performer): r=-0.916, p=0.0005	ANOVA (good vs. poor): p=0.033	
g. (curve: Pearson correlation; good vs. poor:repeated measure two-way ANOVA, two-tailed)	Curve (good performer): r=-0.434, p=0.210	Curve (poor performer): r=-0.300, p=0.400	ANOVA (good vs. poor): p=0.0023	
h. (curve: Pearson correlation; good vs. poor:repeated measure two-way ANOVA, two-tailed)	Curve (good performer): r=-0.878, p=0.002	Curve (poor performer): r=-0.529, p=0.143	ANOVA (good vs. poor): p=0.0035	
i. (curve: Pearson correlation; good vs. poor:repeated measure two-way ANOVA, two-tailed)	Curve (good performer): r=-0.682, p=0.030	Curve (poor performer): r=-0.503, p=0.139	ANOVA (good vs. poor): p=0.278	
Extended data figure 5: (267 neurons of 11 good performer mice, and 267 neurons of 4 poor performer mice)				
a. (curve: Pearson correlation)	Close fields (good): r=-0.540, p=0.107	Close fields (poor): r=-0.768, p=0.009	Far fields (good): r=-0.162, p=0.655	Far fields (poor): r=-0.433, p=0.210
b. (curve: Pearson correlation)	Close fields (good): r=-0.834, p=0.005	Close fields (poor): r=-0.389, p=0.301	Far fields (good): r=-0.113, p=0.773	Far fields (poor): r=-0.528, p=0.144
c. (curve: Pearson correlation)	Close fields (good): r=-0.723, p=0.018	Close fields (poor): r=-0.405, p=0.246	Far fields (good): r=-0.661, p=0.038	Far fields (poor): r=-0.412, p=0.237
d. (Mann-Whitney U test, two-tailed)	Good close vs. far : p=0.102		Poor close vs. far : p=0.319	
e. (Mann-Whitney U test, two-tailed)	Good close vs. far : $p=5.697\times10^{-6}$		Poor close vs. far : p=0.002	
f. (Mann-Whitney U test, two-tailed)	Good close vs. far : p=0.080		Poor close vs. far : p=0.935	
Extended data figure 6: (267 neurons of 11 good performer mice)				

bioRxiv preprint doi: https://doi.org/10.1101/2025.07.30.667696; this version posted July 30, 2025. The copyright holder for this preprint (which was not certified by peer review) is the author/funder. This article is a US Government work. It is not subject to copyright under 17 USC 105 and is also made available for use under a CC0 license.

b. (Mann-Whitney U test, two-tailed)	regression vs. forward within-day shift (Good): p=0.019 (Avg.regression shift: 9.503 cm; Avg. forward within-day shift: 7.798 cm)	progression vs. backward within-day shift (Good): p=8.297×10^{-5} (Avg. progression shift: 10.834 cm; Avg. backward within-day shift: 8.226 cm)	regression vs. forward within-day shift (Poor): p=0.167 (Avg.regression shift: 11.835 cm; Avg. forward within-day shift: 10.309 cm)	progressionvs. backward within-day shift (Poor): p=0.292 (Avg. progression shift: 12.880 cm; Avg. backward within-day shift: 10.497cm)
c. (Mann-Whitney U test, two-tailed)	regression (dataset 2) vsregression (dataset1): p=0.0097 (Avg.regression (dataset2): 10.98cm, Avg.regression(dataset 1): 9.503cm)		Forward within-day shift (dataset 2) vs Forward within-day shift (dataset1): p=0.1396 (Avg. forward within-day shift (dataset2): 7.956cm, Avg. forward within-day shift (dataset 1): 7.798cm)	
e. (Pearson correlation)	r=-0.8032, p=0.0091			
Extended data figure 7: (428 neurons of 8 good performer mice)				
e. (Pearson correlation)	r=0.855, p=0.002			
f. (Pearson correlation)	r=-0.839, p=0.005			
g. (Pearson correlation)	r=-0.892, p=0.0005			
i. (curve: Pearson correlation; bar plot: Mann-Whitney U test, two side)	Close fields (Slope curve): r=0.711, p=0.021	Far fields 1 (Slope curve): r=0.904, p=0.0003	Far fields 2 (Slope curve): r=0.635, p=0.048	
	Close fields (Field center distance curve): r=-0.722, p=0.028	Far Field 1 (Field center distance curve): r=-0.868, p=0.002	Far fields 2 (Field center distance curve): r=-0.789, p=0.012	
	Close fields (Standard deviation curve): r=-0.907, p=0.00003	Far Field 1 (Standard deviation curve): r=-0.890, p=0.0006	Far fields 2 (Standard deviation curve): r=-0.691, p=0.027	
	Close field vs. far field 1 (slope change) : p=0.267		Close field vs. far field 2 (slope change) : p=0.034	
	Close field vs. far field 1 (slope change) : p=0.004		Close field vs. far field 2 (slope change) : p=0.635	
	Close field vs. far field 1 (slope change) : p=3.875×10^{-8}		Close field vs. far field 2 (slope change) : p=2.213×10^{-14}	

bioRxiv preprint doi: https://doi.org/10.1101/2025.07.30.667696; this version posted July 30, 2025. The copyright holder for this preprint (which was not certified by peer review) is the author/funder. This article is a US Government work. It is not subject to copyright under 17 USC 105 and is also made available for use under a CC0 license.

Method

Animals

All animal procedures were performed in accordance with animal protocol 1524 approved by the Institutional Animal Care and Use Committee (IACUC) at NIH/NINDS. Dataset 1[38] contains 8 male and 7 female GP5.3 mice (C57BL/6J-Tg(Thy1-GCaMP6f)GP5.3Dkim/J, JAX stock #028280[39]). All mice were aged 4–8 months old at the time of the experiment. Dataset 2 contains 6 female and 4 male non-carriers of the strain B6; C3-Tg (Prnp-MAPT*P301S) PS19Vle/J (JAX stock #008169[76]) crossed with GP5.3 mice. Mice were 7-10 months old at the time of the experiment. One male mouse and one female mouse were excluded from downstream analysis due to discontinued days in their imaging schedule. Hence in total 8 mice were analyzed here. All mice were maintained on a reverse 12-hr on/off light schedule. Slice recording experiment utilizes 10 male and 10 female wildtype mice aged 3-4 months. Five male and female mice had Channelrhodopsin-2 virally expressed in CA1 and subiculum.

Microprism construction

Microprism construction follows the previously described procedures[38,41,77]. A canula (MicroGroup, 304H11XX) was attached to one side of a circular cover glass (3 mm, Warner Instruments, 64-0720), and a right angle microprism coated with aluminum on the hypotenuse (1.5 mm, OptoSigma, RBP3-1.5-8-550) was attached to the other side of the cover glass. All attachments were performed using UV-curing optical adhesive (ThorLabs, NOA81).

Microprism implantation surgery

All mice received surgeries as described in previous studies[38,41,77]. Mice were anesthetized using a tabletop anesthesia system (VetEquip, 901806). Anesthesia induction was performed with 3% isoflurane and 1L/min oxygen, and anesthesia state was maintained using 0.5-1.5% isoflurane and 0.7L/min oxygen. Surgery was performed on a stereotaxic alignment system (Kopf Instruments, 1900). Mice body temperature was maintained around 37 °C using a homeothermic pad and monitoring system (Harvard Apparatus, 50-7220 F). After anesthesia induction, dexamethasone (2 mg/kg, VetOne, 13985-533-03) were administered by intraperitoneal (IP) injection for anti-inflammation purpose and slow-release buprenorphine (1 mg/kg, ZooPharm, Buprenorphine SR-LAB) was administered subcutaneously for pain relief. Exposed skull will receive antimicrobial wash by Enroflox 100 (10 mg/mL, VetOne, 13985-948-10).

Microprism implantation procedures followed the previous description[38]. A 3 mm craniotomy was centered at 3.4 mm lateral to the midline and 0.75 mm posterior to the center of the transverse sinus (approximately 5.4 mm posterior to the bregma) at the left hemisphere[38]. A durotomy was then performed over the cerebellum. Mannitol (3 g/kg, Millipore Sigma, 63559) was administered by IP prior to the durotomy. The microprism was inserted into the transverse sinus and sealed to the skull with Vetbond (3 M, 1469SB). The headplate was then mounted on the skull opposite the craniotomy, and the prism and headplate were then adhered to the skull with Metabond (Parkell, S396).

bioRxiv preprint doi: https://doi.org/10.1101/2025.07.30.667696; this version posted July 30, 2025. The copyright holder for this preprint (which was not certified by peer review) is the author/funder. This article is a US Government work. It is not subject to copyright under 17 USC 105 and is also made available for use under a CC0 license.

Virtual Reality

A customized virtual reality (VR) setup was used for the experiment, as described previously[38]. Mice were head-fixed onto an air-supported polystyrene ball (8-inch diameter, Smoothfoam) using a mounted headplate. The ball rotated around an axle which restricted its motion to forward and backward rotation, driving the VR environment along linear tracks without rotation. The VR environment was projected onto a dome screen filling the visual field of mice (270° projection). An optical flow sensor (Paialu, paiModule_10210) with infrared LEDs (DigiKey, 365-1056-ND) was used to record motion of the ball, and output the motion signal to a computer via an Arduino board (Newark, A000062). An approximately 4 mL water reward was provided via a lick tube at the reward location via a solenoid. A lick sensor connected to both the lick tube and headplate holder was used to detect mouse licking. A mouse licking the lick tube creates a closed circuit between the lick sensor, the lick tube, the mouse (from the tongue to the skull), the headplate (which directly contacts the skull), and the headplate holder. The solenoid and lick sensor were controlled using a Multifunction I/O DAQ (National Instruments, PCI-6229). Virtual environments were generated and projected using ViRMEn software (Princeton, version 2016-02-12)[78]. Imaging and behavior data were synchronized by recording a voltage signal of behavioral parameters from the VR system using the DAQ. ViRMEn environments were updated at 60 Hz. The DAQ input/output rate was 1 kHz. The synchronization voltage signal was updated at 20 kHz. Final behavioral outputs were matched to the imaging frame rate (30 Hz, see below) for synchronization.

Environments were colored blue and projected through a blue Wratten filter (Kodak, 53-700) to reduce contamination of the imaging path with projected light. Virtual environments were one-dimensional (1D) linear tracks with patterned walls and patterned visual cues at fixed locations. At the end of the track, mice were immediately teleported to the start of the track.

Two photon imaging

Imaging was performed using an Ultima 2Pplus microscope (Bruker). A tunable laser (Coherent, Chameleon Discovery NX) with 920 nm excitation wavelength was used. Laser scanning was performed using a resonant-galvo scanner (Cambridge Technology, CRS8K). GCaMP fluorescence was isolated using a bandpass emission filter (525/25 nm) and detected using GaAsP photomultiplier tubes (Hamamatsu, H10770PB). A 16x water-immersion objective (Nikon, MRP07220) was used with ultrasound transmission gel (Sonigel, refractive index: 1.335985; Mettler Electronics, 1844) as the immersion media.

The anterior-posterior (AP) and the medial-lateral (ML) angle of the prism (i.e., the angle of the surface of the prism along to the AP or ML direction of the mouse) relative to the head-fixed position of the mouse were measured prior to the imaging. The headplate holder and rotatable objective angles were set daily to align the objective with the prism in the AP and ML direction, respectively, such that the objective was parallel to the prism surface. Black rubber tubing was wrapped around the objective and imaging window to prevent light leakage into the objective.

Microscope control and image acquisition were performed using Prairie View software (Bruker). Raw data was converted to images using the Bruker Image-Block Ripping Utility. In dataset 1, Imaging was performed at 30 Hz with 512 × 512 pixel resolution (corresponding to 500

bioRxiv preprint doi: https://doi.org/10.1101/2025.07.30.667696; this version posted July 30, 2025. The copyright holder for this preprint (which was not certified by peer review) is the author/funder. This article is a US Government work. It is not subject to copyright under 17 USC 105 and is also made available for use under a CC0 license.

μm × 500 μm). In dataset 2, dual plane imaging was used to capture two fields of view (FOVs) simultaneously (15Hz each with 512 × 512 pixel resolution, corresponding to 750 μm × 750 μm). Average beam power at the front of the objective was typically 70–115 mW. Imaging and behavior data were synchronized as described above.

Behavior experiment for dataset 1

Behavior experiment paradigm for mice in dataset 1 was described in a published study[38]. After surgery, mice were allowed to recover for 5 days. Mice were then trained in a 1D VR environment (familiar track 1, F1) and their anticipation of reward was quantified by predictive licking (PL) and predictive slowing (PS) metric. PL was measured as the percentage of licks that occurred prior to the reward among those in the whole track, except licks for reward consumption. PS was calculated as the percentile of deceleration prior the reward compared to those in the reset of the track[38]. Mice were regarded as being familiar to F1 if they display stable reward anticipation (less than 5% change in PL and PS) for 3 days.

The mice then performed a 10-day spatial learning task in another 1D VR environment (Novel track, N1), which was a 1000-cm linear track with patterned walls and visual cues as shown in **Fig. 1c**. A reward was placed at the 890-cm position. During experiment, the 15 participating mice were separated into good performers and poor performers based on their PS and PL. PS and PL were calculated with different distance thresholds toward reward (PS: 50, 100, 150 and 200 cm distance threshold; PL: 20, 35, 50 and 75 cm distance threshold) for all 10 days in N1 and averaged across days, generating 8 values (4 from PS and 4 from PL) per mouse. All 15 mice were ranked based on each set of values from high to low. The mice that were consistently ranked in the lower half (i.e., with low performance values) for all 8 sets of values regardless of the chosen parameters were categorized as poor performers. The remaining mice were categorized as good performers.

Behavior experiment for dataset 2

Mice were first trained in a 1 m environment to encourage running. Mice were then trained in a 6m environment (F2) with a single reward in a passive task (i.e., the mouse received a water reward as long as they reached the reward location) until they displayed significant reward prediction behaviors. They were further trained to perform an active learning task in the same track. For this task, mice were required to actively stop (defined as one second with speed less than 5 cm/s) within a 50 cm reward zone to receive a reward. Mice could only receive one reward per run. All mice underwent manual training, consisting of manually stopping the mouse in the reward zone each run, for the initial 3-4 days. If a mouse started to stop on its own, it was further trained without manual stopping with a shortened stop period (0.5s). Once a mouse stopped on its own with the shortened stop period, the full 1s stop period was applied. Additional manual training was performed periodically as necessary to reinforce correct behavior.

Once mice performed well on their own, as measured by a stable percentage of runs receiving reward (success rate), the mice were imaged in the F2 for several baseline days and then imaged in a N2 (6m with new landmarks and a 50 cm reward zone in a different location) for spatial learning and imaging across 10 days. PL was measured for reward prediction within 20 cm before reward delivery location, and PS was measured for reward prediction within 90 cm before reward delivery location. For the runs without reward delivery, PL was measured within 20 cm

bioRxiv preprint doi: https://doi.org/10.1101/2025.07.30.667696; this version posted July 30, 2025. The copyright holder for this preprint (which was not certified by peer review) is the author/funder. This article is a US Government work. It is not subject to copyright under 17 USC 105 and is also made available for use under a CC0 license.

before the end of the reward zone, while PS was measured within 90 cm before the end of the reward zone. Averaged PS and PL across the last six learning days were calculated for each participated mice, and they were compared to the predefined PL threshold (22.2 th percentile) and PS threshold (65.7th percentile)[38] to determine whether the mice is a good performer[38]. The thresholds were obtained from dataset 1 and were the minima of averaged PL and PS of good performers across days 5 to 10 under the same distance threshold. A mouse was defined as a good performer if either of its averaged PL or PS passed the threshold.

Behavior experiment for electrophysiology

Mice were first trained in a 1m environment to encourage running, and then were trained in a 10m environment (FE) with a single reward in a passive task described above. After 3 weeks of training, Mice performed 3-week spatial learning in another environment with different landmark settings and single reward (NE1). Averaged PS and PL across the last six days were calculated for each participated mice, and they were compared to the predefined PL threshold (22.2 th percentile) and PS threshold (65.7th percentile)[38] to determine whether the mice is a good performer[38]

Image processing

Imaging data of dataset 1 was down-sampled by taking the average of each consecutive block of 3 frames and processed as previously described using published MATLAB® scripts[41]. Dataset 2 was similarly processed but by rolling average of consecutive 2 frames. Cross-correlation based rigid motion correction was performed to the imaging data. Identification of region of interests (ROIs) that could represent neuron footprints in dataset 1 was performed using principal component analysis combined with independent component analysis[79]. ROI identification for dataset 2 was performed using Suite2p[80]. To remove artifactual ROIs occasionally caused by light leakage, ROIs with an extreme non-circularity (the aspect ratio of the ellipse best fitting the shape of the ROI $\geq$ 3) were removed from further analysis. The fluorescence time course of remaining ROIs was then extracted. The fractional change in fluorescence with respect to baseline ($\Delta F/F$) was calculated as $(F(t) - F_0(t)) / F_0(t)$[77]. For each cell, significant calcium transients ($\Delta F/F$) were identified using amplitude and duration thresholds, such that the false-positive rate of significant transient identification was 1%[81]. A final $\Delta F/F$ including only the significant calcium transients was used for all further analyses. A speed threshold was further applied to $\Delta F/F$ and was calculated by generating a 100-point histogram of all instantaneous velocities greater than 0 and taking the value twice the center of the first bin (approximately 1% of max positive speed). The $\Delta F/F$ points when the mouse was moving below the speed threshold were excluded from following analyses.

Cross-day common cell identification

With the anatomical footprints of detected cells in each day, we used an established probabilistic modeling methods described in previous studies[38,82] to identify the common cells across days. We first perform automatic pairwise rigid registration of the imaging sessions to identify common neuron pairs between each of the two imaging days. Manual registrations were applied to the imaging session pairs with failed automatic alignment. Pairwise registration results

bioRxiv preprint doi: https://doi.org/10.1101/2025.07.30.667696; this version posted July 30, 2025. The copyright holder for this preprint (which was not certified by peer review) is the author/funder. This article is a US Government work. It is not subject to copyright under 17 USC 105 and is also made available for use under a CC0 license.

were combined to determine the common neuron across all 10 days of the spatial learning task (cross-day neuron). Due to the pairwise nature of the registration, a neuron in a day may be aligned to different neurons in the following days. Manual examinations were applied to these cases to select the optimal alignment results that display the highest level of anatomical footprint consistency across all the last day in familiar track and the 10 learning days in novel track.

Classification of grid cells within a single day

The classification of grid cells within a single day was conducted based on their spatial fields of averaged activities across runs, as reported previously[38,41]. Speed-thresholded ΔF/F for a cell was spatially binned along the track in 5 cm bins and averaged across runs. A gaussian window of 3 spatial bins was applied to the averaged ΔF/F trace to smooth the data. Spatial fields were identified by comparing the amplitude of original smoothed ΔF/F with a random ΔF/F distribution created by 1000 bootstrapped shuffled responses. Each bootstrapped shuffled response was generated by rotating the ΔF/F trace of the whole session so that for every time point of the recording, its track position was preserved but its calcium response was changed. The ΔF/F trace was rotated by starting the trace from random sample numbers chosen from the interval $0.05 \times N_{samples}$ to $0.95 \times N_{samples}$, where $N_{samples}$ was the number of samples in the ΔF/F trace. A shuffled mean ΔF/F was calculated for each rotation. For each 5 cm bin, the p_{value} equaled the percentage of shuffled mean ΔF/F that was greater than or equal to the real mean ΔF/F. Therefore, $1-p_{value}$ equaled the percentage of shuffled mean ΔF/F lower than the real mean ΔF/F. A spatial field was defined as a region of at least 3 consecutive 5 cm bins (except that the fields at the beginning and end of the track could have 2 bins) that had a mean ΔF/F higher than at least 80% of 1000 shuffles at the corresponding bins ($1- p_{value} \leq 0.2$). Additionally, at least 10% of runs were required to have a significant calcium transient in the spatial field.

Grid cells were further identified within each recording day based on the following criteria. (1) Grid cells need to have at least two spatial fields on the track. (2) Each grid cell must have more than L/(5w) transitions between in-field and out-of-field periods, where L represents track length and w represents mean field width of the grid cell's response. (3) The widest field of the response must be smaller than 5w. (4) At least 30% of the bins must be assigned to either in-field or out-of-field periods. (5) The ratio between in-field and out-of-field mean ΔF/F to be ≥ 2[41,53,83].

bioRxiv preprint doi: https://doi.org/10.1101/2025.07.30.667696; this version posted July 30, 2025. The copyright holder for this preprint (which was not certified by peer review) is the author/funder. This article is a US Government work. It is not subject to copyright under 17 USC 105 and is also made available for use under a CC0 license.

Classification of cue cells within a single day

To identify cue cells, cue scores of each cell on a specific day session was first were calculated based on a previously published method[42]. The activity of the cell was first shifted to best match a cue template, which contained ones and zeros representing track areas with and without landmarks, respectively. A landmark zone was identified for each landmark by including the landmark itself and the surrounding region expanded by half of the landmark width on both sides. The correlations between shifted activity and cue template within individual landmark zones were calculated and were further averaged as cue score of the cell. 200 shuffled cue scores were generated for each cell using the same calculation but by randomizing the landmark location in the cue template. Shuffled cue scores from all cells across all FOVs and imaging days were pooled to generate an overall cue score distribution. A cell was identified as a single-day cue cell if its cue score on the day was above the 80th percentile of the shuffle cue score distribution. While previous studies used a $95^{\mathbf{th}}$ percentile threshold[38,41,42], an $80^{\mathbf{th}}$ percentile threshold was used here to ensure the identification of all cells with landmark-matched activity. Cue scores of cells were calculated toward left landmarks, right landmarks and both side of landmarks. A cue cell could be identified based on any landmark type.

Detecting cross-day fields on a run-by-run basis

We developed a four-stage algorithm to more faithfully detect activity fields of grid cells as they could shift on a run-by-run basis and also fail to appear in specific runs ("missed-field runs")[41,83]. In general, run fields were first identified on a run-by-run (RBR) basis (Stage 1). Then we connect individual run fields in neighboring runs into cross-run field segments (Stage 2). Next, we connected the neighboring cross-run field segments into cross-day fields (Stage 3). Finally, we merge the cross-day fields with similar trajectories (Stage 4).

Stage 1. Identifying spatial fields on a run-by-run basis ("run fields").

The speed-thresholded ΔF/F for a cell was spatially binned along the track in 2.5 cm bins to generate a 2D spatial activity matrix representing the cell's activation along track in individual runs. Run fields were identified on a run-by-run basis using the p_{value} based method described above ("**Classification of grid cells within a single day**"). In each run, 1-p_{value} of each bin were calculated by comparing the ΔF/F amplitude within the bin to a random ΔF/F distribution created by 1000 bootstrapped shuffled responses, generated by rotating the ΔF/F trace of the run from random sample numbers chosen from the interval $0.05 \times N_{samples}$ to $0.95 \times N_{samples}$, where $N_{samples}$ was the number of samples in the ΔF/F trace of the run. In-field periods were defined as three or more adjacent bins (except at the beginning and end of the track where two adjacent bins were sufficient) whose 1-$p_{value} \geq 0.85$. Bins with intermediate mean ΔF/F remained unassigned. For dataset 1, only run fields with ≤ 95 cm width were included in the following analysis. For dataset 2, only run fields with ≤ 150cm width were included as the run fields were generally longer in this dataset (**Extended data Fig. 3a, the second panel from the top**).

Stage 2: Connecting run fields in consecutive runs into cross-run field segments

bioRxiv preprint doi: https://doi.org/10.1101/2025.07.30.667696; this version posted July 30, 2025. The copyright holder for this preprint (which was not certified by peer review) is the author/funder. This article is a US Government work. It is not subject to copyright under 17 USC 105 and is also made available for use under a CC0 license.

We connected the centers-of-mass (COMs) of run fields to generate the cross-run field segments. COM of a run field was calculated based on an established method[24]

$$COM = \frac{\sum_i DF_i x_i}{\sum_i DF_i}$$

where DF_i is the ΔF/F of in-run field bin i and x_i is the index of bin i from the start of the track.

For each run field, we searched all the run fields in the next run to identify a closest subsequent run field, a COM of which was within a certain distance range (28 cm for dataset 1, 32 cm for dataset 2, and 10 cm for simulated data).

In the case when there are multiple qualified subsequent fields, we selected the subsequent field with the greatest spatial bin overlap with the current run field. If all subsequent fields had the same level of overlap, we selected the field with the smallest field width. If all remaining subsequent fields had the same width, then we do not connect the current run field with any of the candidates.

In the case when a run field was assigned to multiple run fields in the previous run (preceding fields), we selected the preceding field with the smallest COM distance and largest overlap with the current run field. If all preceding fields had the same level of COM distance and overlap, we selected the field with the smallest field width. If all remaining preceding fields have the same width, then we will not connect the current run field with any of the preceding fields.

After detecting all cross-run field segments by performing the steps above, all other run fields that were not assigned to any segments were kept for the next stage (**Extended data Fig. 3a, the third panel from the top**).

Stage 3: Connecting multiple cross-run field segments to form a cross-day field.

Next, we connected multiple cross-run field segments into cross-day field while permitting runs with missing fields in the middle. For two cross-run field segments, one started earlier (S1) and the other started later (S2) without overlapping runs, we calculated the distances between the averaged COM positions in the last three runs of S1 and the averaged COM positions in the first three runs of S2. S1 and S2 were connected if they showed the closest distance, which was within a distance threshold (28 cm for dataset 1, 38 cm for dataset 2, and 10 cm for simulated data), and the number of runs between the last run of S1 and the first run of S2 that did not have activity (gap) was no more than 7 runs.

In the case where an S1 had multiple qualified S2s, we selected the S2 with the largest number of runs, and the smallest gap toward S1. If multiple qualified S2s still existed after previous selection, we selected the S2 with the smallest track coverage (distance between the most backward COM to the most forward COM). If all S2s displayed the same number of runs, gap size, and track coverage, we did not connect the S1 to any of the S2s.

In the case where an S2 was assigned to multiple S1s, we selected the S1 with the largest number of runs, and the smallest gap toward S2. If multiple qualified S1s still existed after previous selection, we selected the S1 with the smallest track coverage. If all candidate S1s displayed the same number of runs, gap size, and track coverage, we did not connect the S2 to any of the S1s.

bioRxiv preprint doi: https://doi.org/10.1101/2025.07.30.667696; this version posted July 30, 2025. The copyright holder for this preprint (which was not certified by peer review) is the author/funder. This article is a US Government work. It is not subject to copyright under 17 USC 105 and is also made available for use under a CC0 license.

All putative cross-day fields were detected by performing the steps above. Cross-run field segments with more than one run and which were not assigned to any cross-day fields were kept for the following step (**Extended Data Fig. 3a, the fourth panel from the top**).

Stage 4: Connecting cross-day fields sharing similar trajectories

As stage 3 only examined the distance between the ending runs of S1s and onset runs of S2s, a potential problem was that the large field resets between the individual onset and ending runs (e.g., field regression or progression) could make an S2 disconnected with an S1, while their overall trajectories of the two segments were the similar, indicating that they could belong to the same field (**Extended Data Fig. 3a, the fifth panel from the top**). To handle this case, for each of the two cross-run fields from stage 3, one appeared earlier (F1) and the other appeared later (F2) without run overlap, we linearly extended the trend of F1 to the runs occupied by F2, as well as extended F2's trend to the runs of F1. F1 and F2 were connected if (1) the averaged COM location across runs between F1's extended trend line and F2, as well as that between F2's extended trend line and F1, did not exceed a distance threshold (28 cm for dataset 1, 34 cm for dataset 2, and 10 cm for simulated data; (2) the gap between F1's ending run and F2's onset run was no more than 7 runs.

In the case where an F1 had multiple qualified F2s for connection, we selected the F2 with the largest number of runs, and the smallest gap toward F1. If multiple qualified F2s still existed after the previous selection, we selected the F2 with the smallest track coverage. If all candidate F2s displayed the same number of runs, gap size, and track coverage, we did not connect any of the F2s to the F1.

In the case where an F2 was assigned to multiple F1s, we selected the F1 with the largest number of runs, and the smallest gap toward F2. If multiple qualified F1s still existed after previous selection, we selected the F1 with the smallest track coverage. If all candidate F1s displayed the same number of runs, gap size, and track coverage, we did not connect any of the F1s to the F2.

By following the steps above, we constructed the final set of cross-day fields. Only cross-day fields with more than 8 runs were included for the following analysis.

Normalized run field count per run

Since two-photon imaging started after initiating a VR session and ended before terminating the session, there were track areas in the beginning and the end of the session without imaging data. Therefore, run field counts for the first and the last runs were normalized by the length of actual track area with imaging data. For the first run, we excluded the track area at the beginning with at least three consecutive bins without imaging data. For the last run, we excluded the track area at the end with at least three consecutive bins without imaging data. The run field counts were calculated as the number of detected run fields divided by the length of the remaining track for the first and last run. For all remaining runs, the run field counts were normalized by the true track length.

Slope of cross-day fields

For each cross-day fields detected, we performed linear regression analysis (MATLAB® function polyfit) with the COM locations across run-fields, using run indices of run fields as the

bioRxiv preprint doi: https://doi.org/10.1101/2025.07.30.667696; this version posted July 30, 2025. The copyright holder for this preprint (which was not certified by peer review) is the author/funder. This article is a US Government work. It is not subject to copyright under 17 USC 105 and is also made available for use under a CC0 license.

independent variable and COM locations as the dependent variable. The slope thus had the unit cm/run. Strictly non-shifting cross-day fields will have slope = 0. This method is similar to a previous analysis of shifting place fields in CA1 and CA3[24].

Determining shift direction of cross-day fields

We tested the slope of the cross-day field toward the baseline slope distribution generated by bootstrapping the field. We first sorted run fields of each cross-day field based on their COM locations to obtain their possible maximal shifts in forward and backward directions. Then we circularly rotate the sorted run fields and calculated the slope of each rotated fields to construct the bootstrap slope distribution. Two types of cross-day shifting fields were identified:

1. Cross-day backward fields: cross-day fields with slope smaller than or equal to the bottom 10% of the bootstrap slope distribution
2. Cross-day forward fields: cross-day fields with slope larger than or equal to the top 10% of the bootstrap slope distribution.

All remaining fields were regarded as cross-day stationary fields.

Baseline percentage distribution of the cross-day field shift types

To determine whether a shift field type was significantly more abundant than baseline level, for each grid cell we randomized its activity by shuffling run fields within each detected cross-day field 100 times. With the shuffled activity map, we reapplied the cross-day field detection and calculated the fraction of each shift field type. The baseline fraction distribution was constructed from 100 shuffles (**Extended Data Fig. 3e**). The original fraction is considered significantly higher or lower than the baseline if its value is >=95% or <=5% of the entire baseline distribution.

Separating cross-day backward shifting fields to day fields

For each detected cross-day backward shifting field, we isolated its run fields within each day to constitute its day field of that day. Day fields needed to have at least 3 runs to be included in the following analysis. The center of the day fields (along track) was calculated by averaging the COMs of individual run fields of the day fields.

Slope of day fields

Slope of a day field was similarly calculated as "**Slope of cross-day fields**" with the COM locations across the segment's run fields, with run indices of run fields as the independent variable and COM locations as the dependent variable. The slope thus had the unit cm/run.

Field center distances of adjacent day fields

Within a cross-day field, day fields on adjacent days were collected and their centers were calculated as the averaged COM of their run-fields. The absolute distance between the centers of day fields in adjacent days was calculated.

Standard deviation of day fields

Standard deviation of a day field was calculated as the standard deviation of the COM locations of its run-fields.

bioRxiv preprint doi: https://doi.org/10.1101/2025.07.30.667696; this version posted July 30, 2025. The copyright holder for this preprint (which was not certified by peer review) is the author/funder. This article is a US Government work. It is not subject to copyright under 17 USC 105 and is also made available for use under a CC0 license.

Normal distribution of day fields in learning days 7-10

This distribution was constructed by fields that ended within days 7 to 10. A cross-day field appeared between an adjacent landmark pair, containing a back landmark and a front landmark, which was determined based on its onset run field's COM. Its onset distance to the back landmark was calculated as the percentage of the distance between the COM and the back landmark relative to the full distance (100%) between the landmark pair. Its end location within days 7 to 10 was defined as the averaged COM locations of all its run fields within these days. The distance of this end location to the back landmark (identified above for the onset run) was similarly converted to a distance percentage relative to the full distance of the same landmark pair above. This distance, termed "end percentage distance", was negative or positive if the field passed or did not pass the back landmark, respectively.

Similar calculations were applied to all qualified fields, and their combined "end percentage distances" were grouped into 20% bins to obtain the probability density function (PDF). Then the envelope of the PDF was obtained by connecting the counts of each PDF bin. The peak location of the PDF was determined as the peak location of the envelop curve.

The hypothetical normal distribution $N(\mu, \sigma)$ was built such that μ equaled the peak location of the PDF, and σ equaled the absolute range of "end percentage distances" of all fields divided by 6, so that $\mu \pm 3\sigma$ of the normal distribution covered >99% of the range (Empirical rule). With the two parameters, 20000 sets of random values from the distribution were simulated, with each set containing the same number of points as the original "end percentage distance". The PDF of each random set was calculated with the same binning as in the true PDF.

The percentage of day fields passing the back landmark in each track bin were compared to the percentages of the 20000 simulated PDFs at the same location. The percentage of passing fields was defined as significantly smaller than the normal distribution if it was below the bottom 5% of the 20000 simulated percentages, and as significantly larger if it was above the top 95% of the 20000 simulated percentages.

Grouping backward fields to assess their stabilization based on their distances to the back landmarks

For dataset 1 and simulated data, backward fields between two adjacent landmarks were grouped into 2 distance bins based on the distance between their median day field COMs and their back landmarks. For dataset 2, fields were grouped into 3 distance bins. Within each group, the slopes of day fields, the field center distance of adjacent day fields, and the standard deviation of day fields, were calculated using methods described above.

Change in stabilization metrics for close and far fields between early and late learning

To assess the change between the stabilization parameters (the slopes of day fields, the field center distance of adjacent day fields, and the standard deviation of day fields) of early and late learning stages, for each field group (e.g., close or far fields) the averaged parameters of early learning stage (Days 1-3 for dataset 1 and simulated data, Days 1-2 for dataset 2) were subtracted from the parameters from late learning stage (Days 7-10 for all datasets).

bioRxiv preprint doi: https://doi.org/10.1101/2025.07.30.667696; this version posted July 30, 2025. The copyright holder for this preprint (which was not certified by peer review) is the author/funder. This article is a US Government work. It is not subject to copyright under 17 USC 105 and is also made available for use under a CC0 license.

Identification of regression and progression events

All regression and progression events needed to happen between two day fields on adjacent learning days and with any of the two conditions: (1) the earlier day field's last run reached the last run of the day, or (2) the later day field's first run was present in the first run of the day. Both day fields needed to have more than 6 runs (for dataset 1), 3 runs (for dataset 2), and 9 runs (simulated data) to ensure the events were reliably detected. The larger numbers of runs for day fields were used for dataset 1 and simulated data to construct a more reliable "baseline distribution" to reveal their regression events (as described below, "**Significant regression**"), which had smaller magnitudes due to a multi-FOV imaging artifact (dataset1).

Significant regression

A significant regression was determined by comparing the magnitude of the regression with the shift between all adjunct run fields of the pair of day fields that participated in the regression ("baseline distribution"). For dataset 2, the regression magnitude needed to surpass the top 85% of the baseline distribution to be considered significant. The percentile threshold for dataset 1 was 70% due to their significantly smaller magnitudes than those of dataset 2. The percentile threshold for modeling was set to 70% as the simulated environment matches dataset 1.

Co-modular grid cells

Grid modules were identified based on the spacing of the cells of the same mouse[51]. Since grid fields dynamically changed across days, we inspected the activity of each cell on all days (day 0 in F1 and days 1-10 in N1). The spacing of a cell was determined to be its smallest spacing of within-day spatial fields, which were identified based on the $p_{\mathbf{value}}$ of spatially averaged activity across all runs within a day in comparison to shuffles[38,41], as described in " Classification of grid cells within a single day".

To further identify grid modules, we pooled the spacings of all cells from all FOVs in each mouse and identified peaks of the spacing distribution (evaluated by MATLAB® function "ksdensity"). The cells between two valleys were identified as co-modular cells.

Run correlation of co-modular grid cells

For simultaneously imaged co-modular grid cells (with at least 6 cells per module), we first calculated pairwise correlations of the temporal activities of each cell pair in each run. We further calculated the correlation of the pairwise correlations of the exact same pairs on different runs ("run correlation" or "run corr.").

Correlation between trajectories of backward fields at different track distance

The correlation between backward fields was calculated between the segments of two backward fields from different co-modular grid cells that display activations across the same runs. The distance between the two backward field segments were estimated as the distance between the median run field COMs of the two segments. Correlation values were averaged within each 20-cm track bin. Pearson correlation was used to estimate the decreasing trend of the correlation across the track.

bioRxiv preprint doi: https://doi.org/10.1101/2025.07.30.667696; this version posted July 30, 2025. The copyright holder for this preprint (which was not certified by peer review) is the author/funder. This article is a US Government work. It is not subject to copyright under 17 USC 105 and is also made available for use under a CC0 license.

Phase trajectory grid cell population

With a groups of simultaneously imaged co-modular grid cells, we first calculated the power spectral density (PSD) of individual grid cells on each learning day using Welch's method ("pwelch" function in MATLAB®). Grid cell spatial activity was averaged across runs on each day for the calculation. We used WINDOW = hamming (512), NFFT = 2992, NOVERLAP = 461 (~90% overlap between adjacent windows) and Fs=1000 (corresponding to the 1000 1-cm bins on the track) as input parameters to the "pwelch" function. The detrend operation was applied over the resultant PSD from 0.001 cm^{-1} to 0.150 cm^{-1} to reduce low frequency bias (using MATLAB®'s "detrend" function). The top three peaks (f1, f2, and f3) of the PSD were obtained from the "findpeaks" function in MATLAB®. Spatial frequencies of the three peaks were required to be larger than 0.006 cm^{-1} to avoid capturing low-frequency noise. To identify qualified co-modular grid cell populations for the analysis, we first averaged the spatial frequencies of the largest three peaks f1-3 for all cells within a day, and then averaged the results across days, producing a population-level f1-3 (avgf1, avgf2, avgf3) for the three frequency peaks. We included co-modular grid cell populations for further analysis if the difference between avgf3 and the sum of avgf1 and avgf2 was within 8% of avgf3, and the module contained more than 10 grid cells.

The phase of a grid cell on each day was determined based on the spatial frequencies of the three largest peaks (f1, f2, and f3) of its PSD, calculated from its averaged spatial activity across runs on the day. We further performed a 2992-point Fast Fourier Transform (FFT) to its averaged spatial activity. Phases of the FFT were obtained by applying the "angle" function of MATLAB® to the FFT result. The phases of f1, f2, and f3 (φ1, φ2, and φ3, respectively) were determined using the FFT phase at the same spatial frequency.

To visualize the population activity bump within the rhombus, we used an approach similar to a previous study[11]: we first rounded the phase coordinates (φ1, φ2) of each co-modular grid cell to their closest integers, and then binned the coordinates into M*N tiles, where M is the largest value of all φ1s and N is the largest value of all φ2s of co-modular cells, respectively. Each binned coordinate (m, n) thus corresponded to a unique rounded (φ1, φ2). Multiple cells could have the same coordinates due to the rounding. We then sheared the binned coordinates (m, n) into a rhombus shape by:

$$s1 = m + \frac{n}{2}, s2 = \frac{\sqrt{3}}{2} m$$

in which s1 and s2 are the coordinates in the rhombus. We represented each bin by the averaged calcium activity across all grid cells along 5-cm bins along the track, and Gaussian smoothed the activities for final visualization.

To calculate the coordinates of the population-level activity bump, we obtained firing-rate-weighted averages of the phases φ1, φ2, and φ3 across grid cells as functions of track position x:

$$\Psi_{1,2,3}(x) = atan2\left(\sum_{i=1}^{n} \frac{sin(\varphi_{1,2,3}^{i}) r_i(x)}{cos(\varphi_{1,2,3}^{i}) r_i(x)}\right)$$

Here, $r_i(x)$ represents the firing rate for cell i at track location x, and $\Psi_{1,2,3}(x)$ represents the phase coordinates of the population phase trajectory at track location x. We use only $\Psi_{1,2}$ to represent the phase trajectories based on the practice of a previous report[11]. For visualization

bioRxiv preprint doi: https://doi.org/10.1101/2025.07.30.667696; this version posted July 30, 2025. The copyright holder for this preprint (which was not certified by peer review) is the author/funder. This article is a US Government work. It is not subject to copyright under 17 USC 105 and is also made available for use under a CC0 license.

purposes, we unwrapped $\Psi_{1,2}$ using the "unwrap" function in MATLAB® to avoid boundary conditions. Therefore, the population phase trajectory was represented across multiple phase rhombuses.

Distance between population phase trajectories

The distance between the population phase trajectories on adjacent days were calculated as follows: between two trajectories in day n and day n+1, we first corrected phase coordinates of the earlier day's starting point by subtracting or adding 2π if the offset between $\Psi_{1,2}$ of the starting points of the two trajectories were larger than π or smaller than -π. The earlier day's phase coordinates were corrected based on its starting point's correction. We also sheared the trajectory coordinates $\Psi_{1,2}$ to fit a rhombus shape:

$$\Psi_1 = \Psi_1 + \frac{1}{2}\Psi_2, \Psi_2 = \frac{\sqrt{3}}{2}\Psi_2$$

After correction, for each track point with phase coordinates $\Psi_{1,2}$ on the trajectory of day n, we calculated its distance to the point at the same track location ("same-location point") on the trajectory of day n+1. The distances across all track locations were averaged as the distance between the two trajectories.

Rotation of population phase trajectory

To estimate the rotation between two bump trajectories, we subtract the starting point's coordinates from the remaining coordinates of each trajectory to eliminate the effect of lateral shift between two trajectories. Rotation was then estimated from the distance between two trajectories across all track locations.

Lateral shift of population phase trajectory

Lateral shift between start points of trajectories on day n and day n+1 was estimated using the distance between the starting points. Within each grid cell module, shifts between adjacent days were normalized by the averaged shift across all adjacent day pairs.

Phase jumps on the population phase trajectory

To calculate phase jumps, we first calculated the phase trajectories using spatially binned (1 cm) activity within individual runs on each day. We obtained population phase trajectories of individual runs along the track. Each point had coordinates $[\Psi_1, \Psi_2]$. For two points along the track with 1 cm distance, their "phase jump" was calculated as the distance between their phase coordinates $[\Psi_1, \Psi_2]$. To define the within landmark phase jumps, we symmetrically expanded the landmark in both forward and backward directions along the track. For dataset 1 (**Fig. 4**), we expanded the landmark by 35 cm. For simulated data (**Fig. 5**), we expanded the landmark by 5 cm. Phase jumps within the expanded region were included in the "within landmark" jumps. All other phase jumps were considered as "between landmark" jumps. To focus the analysis on the landmark's influence, we removed the jumps between the last two landmarks (863cm to 909cm), which included a water reward location, and 10 cm in the beginning and end of the track to exclude VR teleportation effects.

bioRxiv preprint doi: https://doi.org/10.1101/2025.07.30.667696; this version posted July 30, 2025. The copyright holder for this preprint (which was not certified by peer review) is the author/funder. This article is a US Government work. It is not subject to copyright under 17 USC 105 and is also made available for use under a CC0 license.

Influence index was calculated by subtracting the averaged between-landmark phase jump across runs from the within-landmark jump of each run, representing the general difference of "between landmark jumps" with individual "within landmark jumps".

Modeling

The core architecture of the model follows the recently published Vector-HaSH model[54] — we use a three-layer architecture, with a grid cell layer, a hippocampal layer, and a sensory layer. However, as compared to[54], we modify Vector-HaSH in a few key aspects: the construction of the grid cell network, the learning of hippocampus-to-grid synapses in the memory scaffold, and the addition of a second timescale of plasticity. Below, we describe the construction of the model, including these changes in detail.

Vector-HaSH is implemented through three inter-connected layers:

(1) Grid cells

Grid cells are simulated using a continuous attractor model of grid cells[13]. Neurons are arranged on a lattice of N=128x128 neurons with periodic boundary conditions, with recurrent connectivity W_{GG} in the form of the mexican-hat interaction profile used in Burak & Fiete 2009[13]. The network is initialized by driving the network for 250 timesteps with aperiodic boundary conditions, followed by 250 timesteps with periodic boundary conditions with unidirectional velocity inputs. This initialization protocol ensures that the initial state of the grid network has a stable triangular lattice of activity[13]. Each timestep corresponds to 2 ms. At the start of each numerical experiment, the network is randomly reinitialized, resulting in a random grid phase at the beginning of the track.

(2) Hippocampal cells

The hippocampal layer receives inputs from sensory cells and is bidirectionally connected to the grid cell layer. As in Vector-HaSH, grid cells randomly project onto a hippocampal layer of size N_H =2000 neurons using a projection matrix W_{HG} . The elements of W_{HG} are each independently drawn from a normal distribution with zero mean and unit variance. In Ref.[54], the return projections from the hippocampal layer to the grid layer, W_{GH}, are pretrained through Hebbian learning to map hippocampal states to their corresponding grid states, and they are then held fixed. Here, we introduce a temporal offset in the learning between the hippocampal and grid layer, i.e., W_{GH} associates the grid state $g(t)$ with an earlier hippocampal state, $h(t-\tau)$. We describe this learning in greater detail below.

(3) Sensory cells

Sensory inputs, corresponding to cues observed along the linear track, are encoded as N_S=200 dimensional random vectors, with each element drawn independently from a normal distribution with zero mean and unit variance. Each landmark activates a subset of sensory cells corresponding to left or right cue cells. Boundaries at the start and end of the track are modeled as strong sensory inputs that drive both left and right sensory cell populations. We set the track length (1000 cm) and landmark locations (8 total) to match experimental conditions. These sensory inputs are fed into the hippocampal layer though sensory-to-hippocampus projections, W_{HS}, that are heteroassociatively learned to map sensory states to place states.

The simulated animal navigates a linear track of 1000 cm featuring 8 landmarks placed to match experimental conditions. Motion is modeled over 5000 timesteps per run, with each timestep

bioRxiv preprint doi: https://doi.org/10.1101/2025.07.30.667696; this version posted July 30, 2025. The copyright holder for this preprint (which was not certified by peer review) is the author/funder. This article is a US Government work. It is not subject to copyright under 17 USC 105 and is also made available for use under a CC0 license.

corresponding to 30 ms. The position along the track is updated corresponding to a uniform velocity. This velocity is also fed into the grid cell network, using path integration to update the grid phase. Specifically, the uniform velocity vector along the track maps to an input velocity along the grid cell network at an angle that is randomly drawn from $[0,2\pi]$ and is held fixed for each numerical experiment. In addition to this rotation, time-independent Gaussian noise was injected into both (linear and angular) velocity and sensory inputs at each timestep.

The dynamics of Vector-HaSH proceed as follows:

In the absence of any sensory cues, grid cells path integrated the noisy velocity inputs to update the grid phases at each time step. The updated grid states project onto the hippocampal layer as

$$h(t) = ReLU(W_{HG}g(t) + b)$$

where $ReLU$ is the rectifying function ($ReLU(x) = x$ for $x > 0$ and $ReLU(x) = 0$ for $x \leq 0$), and b is a parameter that controls the sparsity of the hippocampal representations in Vector-HaSH.

At every 20th timestep (corresponding to 40 ms), grid cells receive additional inputs from hippocampal cells

$$g(t) = W_{GH}h(t)$$

The grid cell network runs additional steps of the recurrent network dynamics without any velocity input to arrive at a stable steady state of the continuous attractor network dynamics.

During these hippocampus-to-grid updates, the hippocampus-to-grid synaptic weights are also updated using the temporal delayed learning rule previously described.

Since the grid activations are now continuous valued, we learn the hippocampus-to-grid associations using an online version of pseudoinverse learning[84] instead of Hebbian learning. The weights W_{GH} are thus updated through online pseudoinverse learning to associate $g(t)$ and $h(t - \tau)$. The time delay τ was set to 16 timesteps (corresponding to 32 ms) for all simulations.

To quantify the change in the magnitude of the hippocampus-to-grid synaptic weights, in **Fig. 5b**, we compute the Frobenius norm of the matrix of synaptic weights.

$$\mathrm{norm}(W_{GH}) = \sqrt{\sum_{i,j} [W_{GH}]_{ij}^2}$$

where $[W_{GH}]_{ij}$ refers to the $(i,j)^{th}$ element of the matrix W_{GH}, i.e., $[W_{GH}]_{ij}$ equals the synaptic weight from neuron j in the model's hippocampal layer to neuron i in the model's grid layer. Note that this measure of the magnitude of synaptic weights quantifies how strong the hippocampal-to-grid synapses are, irrespective of their excitatory or inhibitory nature.

At a sensory cue location, the sensory inputs associated with the cue project into the hippocampal layer and update the hippocampal state. This new hippocampal state is in turn used to update the grid state, as well as the hippocampus-to-grid weights:

$$h(t) = W_{HS}s(t)$$

and

$$g(t) = (1 - \gamma_s)\, g(t-1) + \gamma_s W_{GH}h(t),$$

bioRxiv preprint doi: https://doi.org/10.1101/2025.07.30.667696; this version posted July 30, 2025. The copyright holder for this preprint (which was not certified by peer review) is the author/funder. This article is a US Government work. It is not subject to copyright under 17 USC 105 and is also made available for use under a CC0 license.

where γ_s=0.1 is a parameter that controls the strength of the sensory cue information in determining the grid phase in Vector-HaSH. Note that this hippocampal update is implemented at all sensory cue locations, regardless of whether they occur at the periodic hippocampal updates of every 20 timesteps.

Before beginning the numerical simulation in a novel environment, the model is trained on a different environment for 20 runs, with identical track length but randomly drawn sensory cues, to simulate previous learning in familiar environments as in experimental conditions. This initial learning provides the initial conditions for the synaptic weight matrices in the novel environment.

To capture the partial reset of grid fields at the start of each session, we additionally implement a second timescale of the learning dynamics, corresponding to synaptic consolidation. We implement this using two weight matrices: a fast-updating weight buffer that implements rapid online learning using pseudoinverse learning as described above, and a separate slow-updating weight matrix whose elements are given by the fast-updating weight matrix from τ_C=4 runs prior. At the beginning of each new session (i.e., the start of a new day), the fast-updating synaptic weights are re-initialized to the permanent set of slow-updating weights, leading to a partial reset of the learned mappings.

To examine the role of plasticity across trials, we assessed the decoding of spatial position from grid cell population vectors. We constructed a Key Value Memory (KVM) network to learn the association with the grid cell population activity and spatial location. Specifically, we used a 120-cell subsample of the grid cell population activity vector (chosen to evenly sample cells by grid phase) as the keys, and position along the 1000 cm track (binned at 1 cm resolution) as the values. In testing, recall is performed by evaluating the similarity between the query population vector with the stored keys in memory. A softmax is then applied on these similarities, with a large inverse temperature set to 1000, effectively predicting position as the state that has the highest similarity. Additionally, we constrained the predictions of KV network to lie within a 300cm-sized neighborhood of the ground truth, to eliminate any highly discontinuous predictions of spatial location.

The decoding performance was evaluated every five trials using a sliding window: For each evaluation, a block of ten trials was considered for training the Key Value Memory network, which was then evaluated on the subsequent 10 trials. This resulted in 10^4 training samples used to train the network for each evaluation (10 trials, each with 1000 positions). Decoding accuracy was assessed using the mean absolute error (MAE), calculated as the average absolute difference between predicted and true positions. In **Fig. 5p**, we then compared the decoding accuracy for the model as described above, with an equivalent model without any plasticity in the sensory-to-hippocampus and hippocampus-to-grid synaptic weights.

To further examine the role of plasticity, we repeated this analysis in **Extended data fig. 10** with a ten-fold increase in the magnitude of velocity and sensory input noise to accentuate the effect of noise reduction through plasticity. Specifically, we compare the model in three different conditions: (1) With plasticity in the synaptic weights, i.e., as used in the main text; (2) without any plasticity in the synaptic weights; and (3) with plasticity in the synaptic weights, but without any delay in the hippocampus-to-grid associative learning, i.e., setting $\tau = 0$.

Decoding performance of real grid cell data from dataset 1

bioRxiv preprint doi: https://doi.org/10.1101/2025.07.30.667696; this version posted July 30, 2025. The copyright holder for this preprint (which was not certified by peer review) is the author/funder. This article is a US Government work. It is not subject to copyright under 17 USC 105 and is also made available for use under a CC0 license.

Decoding analysis of real grid cell data from dataset 1 was done using the same strategy as the decoding of simulated data mentioned above. Only FOVs with more than 10 cells were analyzed. Spatial activity within the first and last 150 cm of the track were removed from analysis due to unstable behavior[38]. The decoding performance was evaluated every day using a sliding window: for each evaluation, a block of two days was used for training the KVM network, while the subsequent 2 days were used for the evaluation.

Slice preparation

After animals were anesthetized with isoflurane, brain was quickly removed following decapitation in ice-cold solution containing (in mM) 90 sucrose, 80 NaCl, 3.5 KCl, 0.5 $CaCl_2$, 4.5 $MgCl_2$, 24 $NaHCO_3$, 1.25 NaH_2PO_4, and 10 glucose saturated with 95% O_2 and 5% CO_2. For field recordings, horizontal hippocampal-entorhinal slices (300 µm) were prepared with the plate angled around 12 degree so that the caudal portion is raised compared to the rostral portion (ref) using a Leica vibratome 1200S, as described in [55-57]. For whole cell recordings, sagittal slices (300 µm) were prepared with the plate also angled around 12 degrees[55-57] so that the caudal portion is raised compared to the rostral portion. Slices were then warm incubated (~32℃) in artificial cerebrospinal fluid (ACSF) containing (in mM) 124 NaCl, 2.5 KCl, 2.5 $CaCl_2$, 1.3 $MgCl_2$, 26.2 $NaHCO_3$, 1 NaH_2PO_4, 20 glucose, and 0.4 ascorbic acid saturated with 95% O_2 and 5% CO_2 for 20 minutes and then returned to room temperature until recording. For recording, slices were transferred to a submerged recording chamber and perfused at 2 ml/min with ACSF saturated with 95% O_2 and 5% CO_2, which was heated at 32℃ using an inline heater (Harvard Apparatus).

Slice electrophysiology

Field recordings were done using patch pipettes filled with ACSF. Electrical stimulation of subiculum is achieved using bipolar electrodes while resultant fEPSPs signals were recorded from layer II and Layer V entorhinal cortex with two separate electrodes. The slope and amplitude of the fEPSPs were measured. A strong electrical stimulation of 4mA is used to induce a post synaptic response in layer V neurons. After a stable baseline of 15 minutes a high frequency theta burst protocol was applied to induce a long-term potentiation as described in Larson et al.,1986[85]. Herein, the Theta burst protocol is composed of 10 bursts of 4 pulses at 100 Hz repeated 3 times every 20 second. Field potentials were acquired using Multiclamp 700B (Molecular Devices) were filtered at 1 kHz and acquired using Digidata 1550B and pClamp software (Molecular Devices). All data were analyzed using pClamp (Sutter Instruments).

For optogenetic stimulation, whole-cell voltage clamp recordings were measured from layer 2 MEC neurons. Recordings were made using glass electrodes (2.5–3.5 MΩ) filled with an internal solution containing (in mM): 120 Cs-methanesulfate, 10 CsCl, 10 HEPES, 0.2 Cs-EGTA, 4 Na-ATP, 0.4 Na-GTP, 10 phosphocreatine, and 4.4 QX-314 (pH ~ 7.3, ~290 mOsm). 10 µM Alexa Fluo 594 was added to the internal solution to visualize cells after recording. Cells were held at −60 mV for EPSC and 0 mV for IPSC recordings. 10 ms light pulse of 470 nm light from pE-800 (CoolLED) was delivered on layer V every 20 s through 40x lens. Currents from Multiclamp 700B (Molecular Devices) were filtered at 1 kHz and acquired using Digidata 1550B and pClamp software (Molecular Devices). All data were analyzed using Igor Pro 7 (Sutter Instruments). E/I ratio is measured as the ratio between a cell's oEPSC and oIPSC.

bioRxiv preprint doi: https://doi.org/10.1101/2025.07.30.667696; this version posted July 30, 2025. The copyright holder for this preprint (which was not certified by peer review) is the author/funder. This article is a US Government work. It is not subject to copyright under 17 USC 105 and is also made available for use under a CC0 license.

Behavior score

Behavior score for an individual mouse is calculated as follows:

$$Behavior\ score\ = \frac{(PL/100 + \frac{(PS/100 - 0.5)}{0.5})}{2}$$

In which PS and PL are the predictive slowing and predictive licking of the mouse.

oEPSC and oIPSC latencies

Onset latencies of excitatory or inhibitory responses were scored manually using the traces averaged from five traces at each condition. The latencies were calculated from the onset of optogenetic stimulations. Latencies from responses appear after the largest oEPSC and oIPSC level were not selected.

To compare the difference of latency distribution between good and poor performers, for each good and poor performer, the latencies from all individual cells were binned into 2ms bins and the number of latencies were counted in each bin. The latency counts were further normalized by the total counts of each mouse. The difference between the normalized latency counts within each 2ms bin were compared across good and poor performers.

Statistics

Values with error bars are reported as mean ± standard error (SEM). Statistical analyses were described in figure legends. Two-tailed Mann-Whitney U test was used to test the difference between two unpaired value distributions. Two-tailed Wilcoxon matched pairs signed rank test was used to test the difference between two value distributions that constituted paired relationship. Two-tailed Student's t test was used to test the difference between E/I ratio, EPSC and IPSC values. One-sample t-test (two-tailed) was used to test the distribution of regression, progression, and within-day shifts toward standard normal distribution. Repeat measure two-way ANOVA (two-tailed) was used to test the overall difference between two metrics of across different days or runs. Pearson correlation was used to assess the level the increasing (or decreasing) trend of variables across learning. Significance was defined using a p value threshold of 0.05. In figures, we use different numbers of "*" to represent different ranges of p values: * for $p \leq 0.05$, ** for $p \leq 0.01$, and *** for $p \leq 0.001$. "n.s." indicates statistically non-significant ($p > 0.05$). For the analysis in **Fig.3d bottom**, **Fig.1j** and **Fig.5d right** if the percentile ≤ 5% or ≥ 95%, we used "*" to indicate the original value is significantly different from the shuffle distribution. All statistical test results are presented in **Table S1.**

Data availability

All data used in the study will be available upon request to yi.gu@nih.gov.

Code availability

All code used in the study will be available upon request to yi.gu@nih.gov, and will be deposited in a public repository upon acceptance.
